# Traditional and Emerging Maceration Techniques in Red Winemaking: Extraction Mechanisms Shaping Phenolic Composition, Volatile Profile, and Sensory Expression

**DOI:** 10.3390/foods15142571

**Published:** 2026-07-22

**Authors:** Melita Sternad Lemut, Guillaume Antalick, Piergiorgio Comuzzo, Sabrina Voce

**Affiliations:** 1 Wine Research Centre, University of Nova Gorica, Vipavska 13, 5000 Nova Gorica, Slovenia; guillaume.antalick@ung.si; 2 Department of Agricultural, Food, Environmental and Animal Sciences, University of Udine, Via Sondrio 2/A, 33100 Udine, Italy; piergiorgio.comuzzo@uniud.it (P.C.); sabrina.voce@uniud.it (S.V.)

**Keywords:** red wine, cold maceration, fermentative maceration, extended maceration, whole-bunch fermentation, carbonic maceration, cryoextraction, thermovinification, flash release, Accentuated Cut Edges (ACE), pulsed electric fields, ultrasound, high-pressure processing, microwaves, ohmic heating, non-thermal technologies, wine aroma

## Abstract

Maceration is one of the most important tools available to winemakers for shaping the chemical composition, sensory profile, and overall style of red wines. However, understanding the consequences of different maceration choices remains challenging due to the diversity of available techniques, the complexity of extraction phenomena, and the fragmented nature of the available literature, which spans a wide range of technological strategies, cultivars, vintages, winemaking conditions, and analytical approaches. This review thus provides a comprehensive overview of traditional, advanced and emerging maceration techniques used in red winemaking. Conventional approaches, including pre-fermentative cold maceration, fermentative maceration, extended post-fermentative maceration, whole-bunch fermentation, and carbonic maceration, are discussed alongside technological modifications such as cryoextraction, thermovinification, flesh-release and Accentuated Cut Edges, as well as emerging and non-thermal technologies including pulsed electric fields, ultrasound, high-pressure processing, microwaves and ohmic heating. Particular attention is given to the mechanisms governing extraction, with emphasis on how processing conditions and tissue-disruption phenomena influence grape cell wall integrity, membrane permeability, and the release of targeted constituents from different grape tissues. Finally, the advantages, limitations, and practical implications of each technique are highlighted for researchers and winemakers, indicating the need for an integrated consideration of chemical, microbiological, sensory, technological, and economic factors when optimizing red wine production.

## 1. Introduction

Maceration represents a crucial phase in the red winemaking process, with consequences for chemical, physical, and sensory properties of the resulting wines. Currently, traditional maceration techniques remain central to red wine production, enabling the extraction of phenolic compounds and aroma precursors together with nitrogen sources, polysaccharides and minerals from grape skins, seeds and pulps.

Traditional maceration approaches can be categorized into pre-fermentative cold maceration, classical maceration during alcoholic fermentation, post-fermentative extended maceration, whole bunch (whole cluster) fermentative maceration and a unique technique of carbonic maceration. The success of these strategies in extracting key compounds in red winemaking, especially phenolic compounds, is strongly influenced by intrinsic variables, firstly grape variety, environmental conditions, vintage, viticulture practices and harvest time, related to the degree of grape ripeness [1,2,3]. Nevertheless, process factors may further condition the extraction rate while also considering the desired wine style. The weakening of grape cell walls due to enzymatic activities and crushing, duration of maceration, temperature control, sulfur dioxide addition, presence or absence of oxygen, solid/liquid ratio, cap management and agitation all affect the extraction dynamics of grape compounds, also in relation to their location, nature and structure [1,4,5,6,7].

The concept of phenolic maturity includes grape phenolic content and their level of extractability from the grape to the must and wine. In the case of red wines, grape phenolic content often reaches its peak before harvest time, but their level of extractability increases and the composition of extractable grape metabolites evolves in the latest stages of grape ripening [8]. This is mainly due to the evolution of grape cell membrane composition during berry ripening [9]. Therefore, harvest date decision and vintage conditions will particularly impact the outcomes of the different maceration techniques in red winemaking.

Grapes also contain aroma compounds and their precursors that consequentially contribute to the volatile profile of wines. The extractability of aroma precursors is shaped by the same factors as phenolics. In red wine, the aroma maturity is also linked to the phenolic maturity, as non-volatile compounds influence the red wine aroma profile [8,10].

The physico-chemical, enzymatic and microbial activities during maceration and then during fermentation lead to the release and to the formation of important aroma and flavor compounds, as well as exerting an important influence on phenolic polymerization and on the final mouthfeel of the wines [11,12,13,14,15].

The contact of must with the solid part of grape marc first determines the extraction of anthocyanins, together with tannins located in the skin cells. A more prolonged maceration time, especially when alcoholic fermentation occurs, determines, on one hand, a reduction in monomeric anthocyanins content and, on the other hand, a higher concentration of tannins extracted from seeds due to the solubilization of the cuticle caused by ethanol [1,5]. Grape cell constituents pass into the liquid medium by extraction and dissolution, also depending on grape variety, ripeness, and the degree of weakening or disruption of grape berry cells.

The diffusion of extracted compounds throughout the entire must volume is consequently required to facilitate their further dissolution [5,6,7].

During maceration and especially during the following winemaking steps, the phenolic compounds undergo modifications that contribute to color intensity and stability, to the sensory profile and overall wine quality. The formation of anthocyanins–tannins complexes by direct condensation or mediated by acetaldehyde may improve wine color and stability over time, whereas tannins polymerization and their combination with other components (polysaccharides and proteins) influence colloidal stability as well as the sensory profile of wines in terms of mouthfeel, astringency, body and general structure. On the other hand, adsorption phenomena on the solid surface of grape marc during maceration, or on the yeast cell walls during fermentation and aging on lees, might determine a decrease in polyphenols content [16].

Considering the above, there are no rules that allow for determining the best practices to be performed; the modulation of maceration should be based on grape quality and ripeness, but also on the type of wine desired. Generally, shorter maceration is convenient and suitable for young wine, while a more prolonged contact of must with grape marc is often required for higher quality wines with aging potential. In recent years, considerable attention has been directed toward the optimization of maceration protocols. Technological advancements such as cryoextraction, thermovinification, flash release, ACE and the use of inert gases (e.g., CO_2_ or N_2_) have been widely adopted to enhance extraction selectivity and to minimize oxidation and microbial spoilage. Additionally, non-thermal and emerging technologies, such as high-power ultrasound, pulsed electric fields, high hydrostatic pressure, and microwave-assisted extraction, are being actively researched for their ability to accelerate extraction kinetics, to reduce sulfur dioxide usage, and deliver more consistent outcomes across diverse grape batches.

Studies employing advanced analytical techniques (e.g., HPLC, mass spectrometry and NMR) have elucidated the kinetics of grape compounds’ extraction, revealing complex interactions between maceration time, temperature and grape matrix composition [16]. Research also emphasizes the importance of understanding cell wall structure and permeability, as well as the specific localization of bioactive compounds in grape tissues, to rationally design extraction strategies. Furthermore, the integration of different analytical approaches and mathematical modeling is a powerful emerging tool for predicting maceration outcomes and guiding process innovation.

This review aims to provide an exhaustive overview of traditional maceration techniques, as well as to discuss how technological advances have led to greater extraction efficiency, improved process control, and a significant reduction in maceration time while preserving, or even enhancing, wine quality. Thus, the review ultimately provides a comparison of the main outcomes, advantages and limitations of each approach, offering researchers a framework for identifying knowledge gaps and future research needs, while supporting oenologists and producers in evidence-based decision-making on maceration strategies according to grape characteristics, desired wine style, production objectives, and emerging challenges. It also emphasizes the multidisciplinary nature of maceration and the need to continue research in this field, considering chemical, microbial, sensory aspects, and process engineering together. Lastly, as consumer preferences shift toward wines with distinct varietal expression and sustainable production, the evolution of maceration techniques—balancing tradition, technological progress and environmental considerations—will remain a central focus of enological research and practice.

## 2. Traditional Maceration Techniques

In practice, traditional maceration in winemaking relies on three principal approaches: pre-fermentative cold maceration (PreFCM), maceration during alcoholic fermentation, and post-fermentative extended maceration (PostFEM). Maceration during alcoholic fermentation can be performed either with destemmed and crushed grapes (classical maceration, CM+F) or with partial or complete bunch/stems inclusion (whole-bunch fermentative maceration, WBM+F). Since both PreFCM and PostFEM extend skin contact beyond the duration of alcoholic fermentation (AF), they are both generally considered forms of prolonged maceration. In addition, a unique approach, called carbonic maceration, which employs intracellular fermentation inside intact grape berries, has gained increasing attention as a method for shaping wine style.

One of the key factors influencing the choice of maceration technique during winemaking is the intended wine style (Table 1).

### 2.1. Pre-Fermentative Cold Maceration

**Figure 1:**
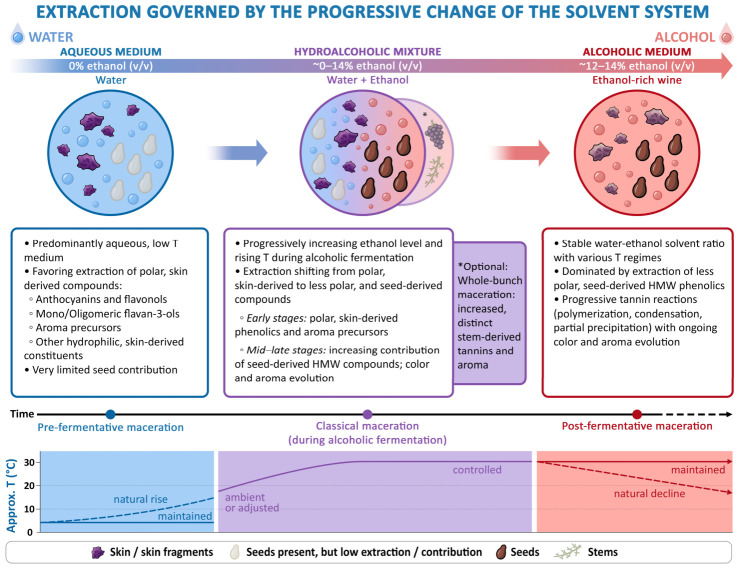
Timeline of hydroalcoholic matrix evolution during traditional maceration processes. Legend: HMW; high-molecular-weight.

Pre-fermentative cold maceration (PreFCM), also referred to as cold soak, is a common red winemaking step in which the grape pomace is kept at lower temperatures prior to AF to modulate wine phenolic and aroma composition. During this stage, skins and seeds remain in contact with the grape juice (must), whose aqueous nature favors extraction of skin-located compounds (Figure 1). Although historically associated mainly with Burgundy Pinot noir wines, the technique is now broadly applied across cultivars and production regions [17,18,19], with its application generally limited to healthy, uncompromised grapes.

PreFCM is generally conducted for 3 to 5 days, although longer durations of up to 10 or even 12 days have also been reported, depending on stylistic objectives and grape characteristics [18,20,21].

The process is typically performed at temperatures ranging from 5 to 15 °C, frequently even lower (3–5 °C) if sufficient enhanced cooling capacity is available [17,22]. Cooling can be achieved using cold rooms, refrigerated tanks, or heat exchangers; however, the use of dry ice (solid CO_2_) is often emphasized as particularly effective, as it enables rapid temperature reduction while simultaneously creating a CO_2_-rich protective atmosphere that limits oxidative reactions in the must [23,24,25].

Maintaining low temperatures during PreFCM delays the onset of AF by keeping yeasts in the lag phase; inhibits the activity of oxidative enzymes, thereby limiting enzymatic oxidation; and helps to delay undesired microbial growth (e.g., acetic acid bacteria), with these effects often supported by the addition of sulfur dioxide [18,26]. In practice, temperature may be maintained or allowed to gradually increase until fermentation begins.

#### 2.1.1. Influence on Phenolic Compounds and Related Sensory Characteristics

PreFCM occurs in an aqueous, ethanol-free medium, with solvent polarity governing phenolic extraction selectivity (Figure 1) [18,27,28].

From a mechanistic perspective, the rapid diffusion of anthocyanins from skin vacuoles promoted under PreFCM conditions can enhance early color intensity and may further support subsequent pigment stabilization and color development through co-pigmentation and polymer formation [29,30,31].

Accordingly, many experimental studies on PreFCM have reported increased levels of **anthocyanins**, related changes in color indices, and, in some cases, higher total phenolic content (TPC), across diverse cultivars originating from various viticultural climates, including Cabernet Sauvignon [32,33], Merlot [22,34], Syrah [22,32,35], Tempranillo [22,36,37], Monastrell [38] and Tannat [27].

However, reported effects on anthocyanins, TPC, and color properties are not always consistent. Some studies show negative or negligible outcomes, with effects that vary across cultivars and vintages or may no longer be evident after AF, with the latter suggesting that the fermentation phase can compensate for initial differences [20,29,37,39,40,41,42,43].

In parallel with anthocyanins, skin-derived low-molecular-weight (LMW) **flavan-3-ols** also increase during PreFCM [27,33,44], with their levels continuing to rise during extended (longer) cold maceration [33,45].

Recent work further supports that the effect of PreFCM on pigments and LMW flavonoids extraction is strongly condition-dependent, with temperature and duration interacting with grape matrix properties determining both the extent and selectivity of extraction [22,45,46,47,48,49].

Collectively, these findings suggest that the variability observed in PreFCM outcomes is not contradictory, but rather reflects the strong dependence of this technique on multiple interacting factors.

Compared to anthocyanins, **flavonols** follow broadly similar extraction patterns, although their lower polarity results in slower extraction kinetics and delayed accumulation in the must [16,18]. However, in Syrah, flavonol concentrations initially increased during skin contact, but later plateaued (dry ice) or decreased (refrigerated grapes) at longer maceration times, suggesting that time-dependent losses of flavonols may be influenced by the cooling strategy applied [23]. Compared to classical fermentative maceration, higher levels of flavonols were reported in PreFCM-treated Tanat wines [27]. Similarly, in a more recent study, higher total flavonol levels were recorded in Teran wines after PreFCM and 13-day traditional maceration, largely attributable to quercetin 3-glucoside and quercetin 3-glucuronide [44]. In contrast, no effect on flavonol extraction was observed in Cabernet Sauvignon [33]. Together, these observations indicate that flavonol behavior during pre-fermentation reflects raw material, protocol, and balance between slower extraction kinetics (duration) and stability processes. Even though they are not directly responsible for red wine color, these pigments can also account for color stabilization and increased color intensity through co-pigmentation processes [50].

Evidence for the behavior of **hydroxycinnamic acids** (HCAs) under PreFCM conditions is limited, with some discussions suggesting that HCA profiles are more cultivar-dependent than vinification method-dependent [51]. Although more studied in white wine, some experimental PreFCM protocols have been associated with a higher presence of HCAs, particularly trans-caftaric acid and total HCAs, compared to fermentative maceration in red wines [27,44]. In contrast, cold soak or its duration had no effect on HCAs in Cabernet Sauvignon after AF [33].

Besides its influence on HCAs, PreFEM was associated with increased levels of **hydroxybenzoic acids** (HBAs) in Teran, including gallic acid [44]. Higher amounts of gallic acid were also detected in Cabernet Sauvignon with longer PreFCM durations, leading to increased concentrations, maintained during wine aging [33].

With regard to **proanthocyanidins**, PreFCM has, in some studies, contrary to general belief, been associated with higher levels of total and seed-derived proanthocyanidins [29,38,52]. This is consistent with the study indicating that proanthocyanidins can also be extracted in the absence of ethanol [53]. In contrast, lower extraction of seed tannins was confirmed in Teran and remained evident in the final wines [54]. Duration appears crucial, as longer PreFCM may enhance seed-derived tannins extraction through increased cell permeability and/or mechanical disruption [33], although extraction remains highly dynamic and compound-specific, with strong interactions between duration, temperature, and grape matrix [25,46,55].

Taken together, available literature on process conditions suggests that in general, longer durations, up to approximately 10 days, tend to be more effective at modifying phenolic composition under typical PreFCM winemaking objectives than shorter ones, while lower temperatures (4–8 °C) achieved by dry ice seem to yield more promising results than moderately higher temperatures [18,26,45,46]. Nevertheless, this should always be confirmed for the specific raw material and desired wine style.

In general, sensory studies associate PreFCM with visual perception of enhanced color intensity and depth, and in some cases, a shift toward more violet or bluish hues [20,22,35], although the effects may vary depending on cultivar, vintage and cooling approach [20,40,56]. Pinot noir, known to be challenged in terms of color intensity and stability due to its naturally low and less diverse anthocyanin content, could be expected to benefit from PreFCM approaches; however, it often shows negative responses [20,42,56].

Regarding mouthfeel, PreFCM has been generally associated with softer, rounder tannins, an improved general mouthfeel, and a temporary reduction in astringency during the early stages of vinification; however, results remain inconsistent across cultivars, vintages, evaluation times, and cryogenic treatments [17,20,22,40,56,57]. In addition, these sensory effects may evolve or diminish during maturation and bottle aging, and their persistence in the final wine remains largely unknown.

Overall, instead of simply increasing phenolic extraction, PreFCM appears to mainly influence when and how different phenolic groups or representatives are released (timing and pattern). When promoting early extraction of key compounds from the skin, this may also influence extraction dynamics at later stages.

#### 2.1.2. Influence on the Aroma Profile: Chemical and Sensory Perspectives

PreFCM may influence wine aroma by enhancing and reshaping the volatile profile through increased extraction of glycosidically bound aroma precursors from grape skins and alterations in grape must nitrogen composition, thereby affecting yeast secondary metabolism [58]. During AF, these precursors are released and transformed into volatile compounds, potentially increasing levels of specific volatiles in the final wine. The extent and direction of this effect depend on grape material (variety, maturity), grape microbiota present, process conditions, and subsequent yeast activity [59,60,61]. In addition, PreFCM-related increased phenolic content may modify aroma perception through matrix effects, including binding interactions and modulation of volatility [62,63].

PreFCM has been reported to increase several aroma compounds, particularly esters and/or acetates in Monastrell [64], Merlot [34], Teran [54], and Tempranillo, Merlot, and Syrah [22]. Additional effects include increases in higher alcohols (isobutanol and isoamyl alcohols) in Monastrell [64], 2-phenylethanol in both Monastrell [64] and Merlot [34], C6 compounds in Teran [54], and, under industrial conditions, norisoprenoids and ethyl esters of straight-chain fatty acids in Shiraz and Nero d’Avola following only one day of cold maceration [65]. A recent study further confirmed a strong cultivar dependence [22].

Notably, after one year of bottle aging, Tempranillo wines showed increased levels of ethyl decanoate, 2-phenylethyl acetate, and decanoic acid, but only when PreFCM was performed using dry ice, suggesting that the cooling approach itself may influence the final aroma profile [43].

Regarding PreFCM duration and temperature, Syrah wines macerated for 72 and 120 h exhibited significantly higher aroma concentrations than those macerated for 24 h [45], whereas wines macerated at 5 °C exhibited higher ester levels than those processed at 10 or 15 °C [66].

Despite considerable variations in experimental protocols and grape cultivars, PreFCM generally favors the release or formation of aroma compounds associated with fruity and floral sensory profiles. Thus, in line with compositional changes, wines produced with PreFCM are often described as fruitier, showing more pronounced fresh fruit notes such as blackcurrant, blackberry and other small red berries, occasionally accompanied by floral nuances, or greater overall aroma intensity [22,45,57,67]. Similar observations have also been obtained for odor activity value calculations [54,68].

Although our understanding of aroma compounds and their precursors continues to improve, translating compositional changes during PreFCM into final wine aroma outcomes remains challenging. New studies focused on volatile markers [69] may help to address this complexity and potentially improve the prediction of flavor development.

In overall summary, a substantial body of valuable research has been conducted on PreFCM, yet the results, particularly those of phenolics, do not offer generalizable models that would allow reliable expectations based on known grape characteristics. This is partly due to limited comparability, with notable differences in experimental and analytical approaches further complicated by cultivar-specific responses and by variation in viticultural management, geo-climatic conditions and harvest timing. At the same time, the technique itself is not strictly defined. What is referred to as PreFCM can vary considerably in research and practice, and this variability is reflected also in the reported outcomes.

### 2.2. Classical Maceration During Fermentation

Classical maceration with fermentation (CM+F), also referred to as fermentative maceration, represents a central and most commonly used extraction step in red winemaking and is also increasingly used for more complex, macerated white wines.

During this process, following destemming and crushing, grape solids remain in contact with the fermenting must, with maceration and AF occurring largely simultaneously, except for the relatively short period before active fermentation begins, corresponding to the yeast lag phase. Depending on the vinification protocol, this step may begin either directly or after selected pre-fermentative treatment [7,70].

In contrast to PreFCM, CM+F takes place during AF in a progressively evolving hydroalcoholic system (Figure 1) where rising temperature further enhances phenolic extraction by promoting cell wall permeability, and carbon dioxide and sulfur dioxide may influence the process by altering the physical and chemical environment [17,20,42,71,72].

In this context, CM+F represents a key stage in determining wine color, taste, and structure, while also contributing to the development of the aroma profile through the extraction and yeast-mediated transformation of aroma-active compounds [16,42,61].

Maceration coupled with AF usually lasts around 7–14 days, with the duration varying depending on temperature, yeast strain, sugar concentration, cap management and the desired wine style [7,70,73,74]. Contact time between grape solids and the fermenting must governs particularly phenolic extraction and related sensory properties, with longer durations generally enhancing extraction; however, in a class-dependent manner [16,17,74]. The optimal maceration length depends largely on intended wine style. A shorter maceration (7–9 days) may better preserve freshness and drinkability in wines intended for early consumption, whereas longer maceration (up to 11 days) may favor the extraction of phenolic compounds associated with greater color stability and structure in wines intended for aging [73].

Fermentation temperatures in red winemaking are usually maintained between 20 and 30 °C. Higher temperatures tend to enhance phenolic extraction, but they may also accelerate degradation processes or lead to the loss of volatile compounds, particularly low-boiling ones, due to increased volatilization [75]. Temperature management is thus crucial in balancing extraction efficiency with the preservation of aroma [16,47,75,76].

In addition to CM+F duration, temperature, chemical factors (e.g., solvent composition), and biological factors (e.g., yeasts), physical factors such as cap management also play an important role in shaping the final wine composition [17].

During CM+F, continuous or periodic cap management is needed to maintain effective solid–liquid contact and ensure homogeneous fermentation conditions. As carbon dioxide is produced during fermentation, grape skins are lifted to the surface, forming a dense cap. Within this layer, yeast activity is often enhanced by local nutrient availability, and temperature can increase more rapidly than in the underlying fermenting juice, with differences of up to 14 °C [72].

If left unmanaged, temperature gradients may develop within the cap, which can enhance extraction but also negatively affect yeast performance if excessive. At the same time, a compact or poorly mixed cap can limit extraction from inner layers that are not in direct contact with the must as well as increase susceptibility to oxidation at the surface [7,17,74,77].

For these reasons, cap management is routinely carried out, typically once or twice per day. In practice, this is done using a few main approaches or their combinations. The most common are punch-down (pigeage), where the cap is manually or mechanically broken and pushed back into the liquid, and pump-over (remontage), where liquid is drawn from the bottom of the tank and sprayed over the cap from above. While punch-down is often used at a smaller scale, pump-over tends to be more common in larger tanks. Other approaches include the use of baffled rotary tanks, which facilitate the submergence of grape solids into the liquid phase [72], and racking (délestage), a more intensive cap management technique involving complete drainage of the must followed by its return over the cap. The temporary collapse of the pomace during this process improves mixing and promotes extraction and migration into the liquid of skin-derived compounds [17]. Recently, less intensive (“soft”) approaches have also been explored. The aim is to manage the cap without strong mechanical action, for example by using air injection or controlled release of CO_2_ to keep the cap loose and improve contact with the liquid phase [78].

Because cap management operations influence not only phenolic extraction, but also oxygen exposure and temperature distribution within the fermenting mass, they contribute substantially to the overall control of fermentation [7,17,42,74,79]. The extent to which these practices influence extraction depends on cap management intensity, timing, and duration, while their impact may also differ considerably among cultivars and grape matrices [80].

In practice, CM+F is a very flexible approach in which key parameters can be tailored to achieve different styles ranging from lighter, fruit-driven wines to more structured, tannin-rich wines. At the same time, CM+F is not only an extraction phase but also a phase of active transformation, where phenolic compounds undergo oxidation, polymerization, and interactions that can influence color parameters and mouthfeel, while in parallel yeast-driven and chemical transformations contribute to the development and evolution of wine aroma.

#### 2.2.1. Influence on Phenolic Compounds and Related Sensory Characteristics

During CM+F, phenolic compounds are extracted primarily from grape solids, beginning with an initial rapid release from disrupted cells at crushing, followed by a slower, diffusion-driven process governed by solvent penetration, solute dissolution, and transfer from the solid matrix into the surrounding must (Figure 1) [72].

Given the defining role of anthocyanins and tannins in red wine style and quality, CM+F is fundamentally designed to extract and modulate both; however, their extraction kinetics differ markedly, as does the behavior of skin- and seed-derived tannins [7,16,26,74,81]. These differences are largely driven by their cellular localization, interactions with cell wall components, and intrinsic molecular characteristics, such as size and polarity [16,74].

Monomeric **anthocyanins** are typically extracted rapidly during the early stages of AF, generally reaching maximum concentrations within the first 4–7 days of maceration. Following this initial peak, their concentrations often decline, sometimes substantially. This pattern has been consistently observed across a range of cultivars, including Cabernet Sauvignon, Merlot, Syrah, Pinot noir, Monastrell and Aglianico [19,34,71,73,76,81,82,83,84,85]. The decline can be attributed to processes such as adsorption onto yeast and solids, incorporation into polymeric pigments, and chemical degradation [16].

Maceration duration primarily affects anthocyanin composition and stability, rather than simply their concentration. While longer maceration does not necessarily result in higher anthocyanin levels, it may promote their transformation into more stable forms, including polymeric pigments, and enhance interactions with other phenolic compounds [26,56,76,86,87].

Temperature plays an important role by influencing cell membrane permeability in grape solids and enhancing phenolic solubility, thereby promoting diffusion and accelerating extraction [81,88]. Higher temperatures generally result in greater early anthocyanin concentrations, but can also promote degradation, oxidation, and polymerization [42,72].

In a recent study, fermentation temperature was identified as an important factor governing anthocyanin content in Pinot noir, whereas cap management had a lesser effect [47]. In contrast, temperature alone showed limited influence on anthocyanin extraction or degradation during Nebbiolo wine-like extraction and lab-scale winemaking [76]. Another study evaluated the impact of seeds on anthocyanin extraction in four red grape varieties (Aglianico, Nebbiolo, Primitivo, and Sangiovese) in 10-day model macerations, showing that seed presence did not significantly affect anthocyanin levels in solution, although it generally promoted polymerization, while anthocyanins retained by seeds appeared to be cultivar-dependent [89].

Due to their water solubility, **flavan-3-ols** from skins are extracted rapidly, typically reaching 80–85% of their maximum concentration within approximately five days. Conversely, seed-derived compounds require longer maceration for ten days or more [5,16,73,81,85,90,91]. Consequently, initial wines feature skin-derived monomers and oligomers, whereas prolonged maceration increases the contribution of seed-located compounds, particularly their galloylated forms [71,90,92,93].

**Flavonols**, characterized by lower polarity and scarce hydrophilicity, are rapidly extracted during early stages of fermentative maceration, although at a slower rate than anthocyanins [81]. Their extraction generally increases over the first 5–7 days of fermentative maceration, is further enhanced after 8–9 days, and may later decline due to the hydrolysis of unstable aglycones [16]. In a recent study on Pinot noir, quercetin glucosides predominated during CM+F, while their relative abundance, particularly that of quercetin-3-glucoside, decreased during aging. The same authors reported that increased fermentation temperatures resulted in higher total flavonols, whereas cap management had a comparatively smaller effect [47]. Beyond their role in copigmentation and wine color stabilization, flavonols have been linked to the perception of a more velvety astringency [47,94].

Relatively few studies have specifically addressed the behavior of **phenolic acids** during fermentative maceration in red wines. Theoretically, HCAs are expected to be extracted early due to their relatively high polarity and distribution not only in the skins but also in the pulp. In line with this, cinnamate concentrations in Cabernet Sauvignon peaked around day 4 irrespective of fermentation temperature, followed by a marked decline after maceration, likely reflecting their susceptibility to enzymatic oxidation [81]. However, increasing levels of caftaric and coutaric acids were reported in Monastrell up to 10 days of maceration, also noting that these compounds act as important substrates for polyphenol oxidase (PPO), while emphasizing that enzyme activity is largely confined to the early stages of vinification [95]. Similarly, concentrations of phenolic acids, both HBAs and HCAs, increased with maceration duration, suggesting that longer maceration may lead to higher levels of these compounds in red wines [96]. The evolution of some key HBAs (e.g., gallic, protocatechuic, and vanillic acids) during CM+F is often linked to the hydrolysis of ester-bound or glycosylated forms and, to some extent, the breakdown of more complex phenolics; however, direct extraction from grape skins and seeds remains a significant source of these compounds [7,60,90].

The extraction behavior of **proanthocyanidins** is primarily governed by their relatively high molecular weight (HMW) and limited solubility in aqueous media, together with structural constraints within the grape seeds [7,17,60]. In particular, the outer lipidic cuticle acts as a diffusion barrier, often resulting in an initial lag phase that is progressively overcome as ethanol concentration increases during AF [17,53,60]. Nevertheless, ethanol is not strictly required for their extraction, as release can also occur under aqueous conditions; however, less efficiently [53]. In Cabernet Sauvignon, total tannin concentration peaked around day 9 of maceration [85]. As oligomer size increases, extraction becomes slower, and the effective retention of HMW proanthocyanidins in wine may also be reduced [16,90]. Their extraction and subsequent evolution are also influenced by the wine matrix, particularly through interactions with anthocyanins, mannoproteins, and polysaccharides [16].

The literature generally supports a progressive shift from skin- to seed-derived tannins during CM+F [90,97] as illustrated in Figure 1. This behavior has been consistently observed in cultivars such as Monastrell, Cabernet Sauvignon, Merlot, and Syrah, although both concentration levels and extraction magnitude vary among varieties [98,99,100]. Nonetheless, the addition of grape seed proanthocyanidins of various degrees of polymerization (mDP 1.31–4.63) to Cabernet Sauvignon wines, bottle-aged for 6 months, confirmed the importance of seed tannin polymerization degree on the development of wine chemical and sensory characteristics [101]. Low-mDP seed tannins accelerated tannin maturation and color evolution toward more mature tones together with brick red hue development, whereas high-mDP seed tannins led to greater tannin stability [101].

In addition to time, other process conditions further modulate tannin extraction. Fermentation temperature strongly influences tannin concentrations, with higher temperatures generally leading to increased extraction while having limited effects on structural characteristics. In contrast, cap management appears to exert a comparatively smaller effect on total tannin levels but significantly influences tannin structure, including molecular mass and degree of polymerization [47,72]. Thus, structural parameters of tannins, such as mDP, but also percentage of prodelphinidins (%PD), and degree of galloylation, also evolve during maceration [16,90,98] with a recent study highlighting marked varietal differences [55]. However, despite the progressive increase in seed-derived tannins during CM+F, final wines tend to exhibit a more balanced proportion of seed- and skin-derived tannins than wines subjected to extended maceration [102].

Sensory studies focusing on phenolic-related attributes often report differences in color perception and mouthfeel when CM+F is compared with prolonged maceration approaches (PreFCM and PostFEM).

Regarding color, compared with PreFCM, CM+F wines exhibited lower color intensity and hue in Sangiovese [57] and less ruby and purple tonalities in Teran [44]. However, opposite trends have been reported for Pinot noir CM+F wines, showing higher perceived color intensity, whereas in the same study Barbera d’Asti and Cabernet Sauvignon wines exhibited lower color intensity than PreFCM wines [20]. When compared with PostFEM, CM+F Merlot wines showed more pronounced red reflections and lower perception of brown hues, with differences becoming more evident at higher alcohol levels [102].

Effects on mouthfeel are often reflected in astringency and, to a lesser extent, in bitterness and tannin perception. A clear cultivar-dependent response to increasing maceration duration (5, 10, and 20 days) has been reported, although it remains unclear whether all treatments were conducted within the active fermentation phase [100]. Under their experimental conditions, perceived astringency increased progressively in Monastrell and Cabernet Sauvignon wines, whereas no significant changes were observed in Syrah. Bitterness was generally less affected, except in Cabernet Sauvignon, where its perception decreased following the longest maceration treatment. With regard to the mDP of seed proanthocyanidins, a higher proportion of low-mDP seed tannins might be linked to a more velvety mouthfeel, whereas a greater proportion of high-mDP seed tannins may contribute to a harsher, more astringent mouthfeel [101]. In Teran wines, only minor differences in perceived astringency and bitterness were observed among treatments, although CM+F wines scored lower for tannin presence and tannin quality than wines produced using prolonged maceration and saignée combinations [44].

Besides maceration duration, temperature may further influence mouthfeel, with higher fermentation temperatures associated with increased perceived astringency and bitterness in Cabernet Sauvignon wines [103].

#### 2.2.2. Influence on the Aroma Profile: Chemical and Sensory Perspectives

The influence of CM+F on aroma is not always easy to interpret. In most cases, volatile formation is primarily driven by yeast metabolism and fermentation conditions such as temperature, nutrient status, and oxygen regime [59,61], while maceration contributes mainly through matrix modification and extraction of aroma precursors including amino acids that can influence yeast metabolism and, in turn, wine volatile composition. At least 27 relevant aroma compounds derive from grape-specific precursors, including glycosides, glutathionyl and cysteinyl conjugates, and other non-volatile molecules, with some further formed during fermentation and others accumulating more slowly during aging [59]. In the context of precursor contribution, and compared with pre-fermentative conditions, ethanol also plays an important role as a solvent, enhancing the solubility of various compounds, including more lipophilic aroma molecules and their precursors [17].

Given the dominant role of yeast metabolism in aroma formation, relatively few studies have focused specifically on the effects of CM+F. Existing work has primarily examined maceration duration, with additional influences often arising from associated practices such as cap management. Increasing concentrations of alcohols, esters, and fatty acids were observed between the fourth and seventh day of Vranec maceration [104]. In Karaoğlan wines, comparison of 5, 10, and 15 days maceration periods showed that free aroma compounds reached their peak after 5 days of maceration, whereas glycosidically bound aroma compounds peaked after 15 days [105]. Furthermore, total volatile content peaked after 11 days of Cabernet Sauvignon fermentative maceration, with the authors further suggesting that moderate maceration may favor aroma accumulation, whereas excessive maceration may negatively affect aroma quality [85]. Teran wines likewise exhibited shifts in the volatile composition across different maceration regimes, with shorter treatments (7–10 days) generally associated with higher concentrations of most analyzed aroma compounds [106].

Experimental studies further indicate that cap management can influence volatile composition. Earlier work demonstrated that different cap management regimes altered the concentration of all but five detected aroma compounds with clear cultivar specifics [80]. Similarly, in Barbera, cap management affected the volatile profile after aging, with floating-cap wines showing higher concentrations of C6 alcohols and generally higher levels of esters and acetates than submerged-cap wines, whereas some fatty acids exhibited the opposite trend [107]. Recent studies upgraded these observations, indicating that contrasting process-related redox conditions may result in differences in ester concentrations after aging [108], while a tendency to higher concentrations of esters and terpenes has been observed in Syrah submerged-cap wines compared to the other treatments [109].

Sensory effects of CM+F on odor/aroma are initially influenced by grape variety and overall fermentation conditions. Furthermore, the non-volatile matrix of red wine plays an important role in aroma perception [10]. Thus, maceration duration may influence aroma expression not only through changes in volatiles but also through modifications of phenolics and polysaccharides. Studies comparing CM+F with prolonged maceration regimes frequently report differences in fruity, floral, spicy, and reductive notes, and sometimes in flavor intensity, complexity, or overall aroma balance [26,57,105,106].

In conclusion, despite its crucial role in red winemaking, CM+F is often studied indirectly as a reference treatment (control) when testing alternative maceration techniques with various technological aims and conditions. Consequently, CM+F is rarely evaluated against a consistent baseline, making it difficult to isolate and assess its own specific effects. As a result, current knowledge of CM+F is somewhat fragmented. To address this, a more focused experimental revisiting of CM+F is warranted.

At the same time, reducing matrix-related influences may provide additional clarity. Model systems, such as simulated grape juice, can help isolate specific processes, but also highlight the need for their further development to better reflect key features of the grape matrix, while still acknowledging their limitations.

Table 2 provides a condensed comparison of the key chemical and sensory impacts of traditional maceration techniques and their modifications, while a more detailed summary overview of their technological features, advantages and limitations is provided in the Supplementary Information (Table S1).

### 2.3. Whole Bunch Fermentative Maceration

Besides the classical approach based on destemming and crushing prior to maceration, whole bunch (or whole cluster) fermentative maceration (WBM+F) has gained increasing attention in red winemaking due to its potential to modify wine long-term color stability, aroma, flavor, texture or overall complexity [39,110,111]. The technique involves fermenting intact grape bunches together with their stems (Figure 1), partially or fully replacing destemmed/crushed grapes, with whole bunch inclusion typically ranging from 10 to 100% [110,111,112]. It also promotes partial intracellular/carbonic maceration within intact berries [113], together with enhanced extraction of stem-derived compounds [111,114]. WBM+F is most commonly used and studied in Pinot noir, but it can also be applied to other red grape cultivars.

#### 2.3.1. Influence on Phenolic Compounds and Related Sensory Characteristics

Phenolic-related effects are mainly linked to the extraction of stem-derived **flavan-3-ols** and **flavanonols** such as astilbin, which can increase total tannin concentration and modify their composition compared to fully destemmed fermentations, reflecting the distinct nature of stem-derived compounds compared to those originating from skins and seeds [115,116]. Regarding **anthocyanins**, improved anthocyanin concentration and color intensity balance were observed with 75% compared to 50% whole bunch addition in Primitivo wines [117], whereas no significant effect on anthocyanin levels was found in Pinot noir wines with low bunch inclusion (20%) [39]. Furthermore, in Pinot noir, higher proportions of whole bunches (60–100%) significantly increased the concentrations of **flavan-3-ols** (catechin, epicatechin, gallic acid), several **HCAs,** including caftaric acid, cis-coutaric acid and caffeic acid, and also resveratrol levels in the resulting wines [111]. A following study further confirmed that 60% whole bunch inclusion can increase the proportion of stem- and skin-derived **tannins**, while reducing the relative contribution of seed-derived tannins in the wines [112]. Some of these compounds enriched by WBM+F may also contribute to copigmentation and color stabilization [118]. However, stems may also adsorb anthocyanins, potentially reducing color intensity [117]. In line with the results of chemical analyses, increased astringency and possibly bitterness are reported, but also enhanced sweetness, likely related to the high astilbin content in stems, indicating the potential for modifying mouthfeel and texture complexity [39,114,115].

#### 2.3.2. Influence on the Aroma Profile: Chemical and Sensory Perspectives

Regarding volatile profile and related sensory impacts, whole cluster inclusion in Pinot noir wines was associated with enhanced aroma diversity, including floral, bitter almond, spicy/clove, vegetal, and cooked fruit notes [39,114]. WBM+F may further increase aroma complexity through partial carbonic maceration occurring within intact berries [113]. However, higher stem inclusion may also increase methoxypyrazines, some C6 compounds, β-damascenone, eugenol, guayacol and ethyl cinnamate—compounds associated with vegetative/green, woody, spicy, or “stemmy” sensory notes as reported in the case of Pinot noir or Cabernet Sauvignon [111,119].

Although some specific trends can already be observed to date, research on WBM+F remains relatively limited and fragmented. Additional, more detailed studies involving a wider range of cultivars, stem maturities, inclusion rates, and process conditions are still needed to better understand its chemical, sensory, and aging-related impacts, as well as variability of responses among grape varieties and vintages.

### 2.4. Carbonic Maceration

Carbonic maceration (CM) is a unique winemaking approach in which intact, undamaged grape clusters, usually with stems, are gently placed into a sealed pressure-resistant vessel and initially exposed to an artificially introduced carbon dioxide atmosphere (achieved by gas or dry ice), which is later reinforced by naturally produced gas in the process [79,113,120,121]. CM relies on the ability of whole grape berries to adapt to an oxygen-depleted, CO_2_-rich environment, which almost immediately triggers a metabolic shift within each berry from respiration to fermentative anaerobic metabolism [113].

This process, invented in 1934 by Michel Flanzy, can last from several days up to about 3 weeks—typically between 5 and 15 days—depending on factors such as variety, temperature and desired wine style [17,113]. In general, higher temperatures (around 30–32 °C) shorten maceration to approximately 5–8 days, whereas lower temperatures (e.g., 15 °C) may prolong the process to 15–20 days [113].

In some cases, a small portion of crushed grapes or must is added; however, the weight of the grapes alone also releases some juice that accumulates at the bottom of the vessel and initiates conventional AF in the liquid phase [113,122].

The hallmark of CM is intracellular fermentation occurring inside grape berries. Under anaerobic conditions, grape enzymes initiate sugar metabolism within berry cells, producing small amounts of ethanol (around 1.5–2%) even before yeast-driven AF begins [17,113]. After a certain period, the grapes are crushed (or pressed directly at this stage), and AF is completed either with indigenous or commercial yeast [42].

Its success largely depends on preventing any prior damage or infection of the grape berries. Moderate sulfiting and sometimes bottom-must acidity adjustment is recommended to suppress the activity of acetic acid bacteria or other spoilage microorganisms [17,123].

CM was first implemented in France’s Beaujolais region, which remains the best-known origin for this style of red wines. Today, the technique is widely applied worldwide and can be used not only for red and young wines but also in the production of rosé, fortified, and even sparkling wines [113].

#### 2.4.1. Influences on Phenolic Compounds and Related Sensory Characteristics

Compared with conventional controls, a tendency toward lower anthocyanin and TPC levels may be observed in CM wines, with some differences among studies likely reflecting ethanol formation within intact berries and cultivar-dependent skin toughness [124,125]. Accordingly, lower **anthocyanins**, polymeric pigments and/or TPC levels were observed in Syrah [19], Cascade [126] and Tempranillo and Graciano [127]. In the latter study, CM wines also exhibited lower **flavonol** concentration, but higher **flavanol** and **HCAs** levels in free-run wine, likely due to stem presence during fermentation [127]. In contrast, CM Tempranillo wines showed greater color intensity, higher phenolic and polymeric pigment contents and higher concentrations of vitisins A and B, **pyranoanthocyanins** associated with improved long-term color stability [128].

In line with analytical trends, CM wines often display lighter color and body, tannin mouthfeel and lower astringency compared to red wines made by CM+F [17,42,113,123,125].

#### 2.4.2. Influence on the Aroma Profile: Chemical and Sensory Perspectives

Compared with conventional destemming/crushing or whole bunch fermentation, CM wines are often linked to fruitier and more intense aroma profiles, associated with soft red fruits, e.g., strawberry/cherry, and candy-like notes [123,129,130]. The combination of elevated CO_2_ and ethanol formation within berries likely played a major role in aroma modifications contributing to increased total esters and acetates in Tempranillo [128], and higher concentrations of ethyl dihydrocinnamate, 3-mercaptohexanol and ethyl cinnamate in Grenache and Carignan [131], with ethyl cinnamate previously detected also by Versini et al. [132]. Notably, it has been demonstrated for other plants that the deprivation of oxygen in the atmosphere caused accumulation of trans-cinnamate, which is a key metabolite in the central phenylpropanoid pathway [133].

Interestingly, a recent study explored CM also as a possible tool to achieve lower alcohol wines [134]. In addition, CM approaches may help reduce naturally high malic acid concentrations and are sometimes used for blending purposes [123].

However, despite its growing popularity in both classical and modified versions (semi-carbonic), studies on CM remain relatively scarce, particularly in recent years, where modern analytical approaches combined with well-designed sensory studies could provide important new insights into its overall chemical and sensory impacts across cultivars and vinification conditions. In addition, more standardized experimental protocols with consistent reference treatments are needed to improve comparability among studies.

### 2.5. Post-Fermentative Extended Maceration

Post-fermentative extended maceration (PostFEM) refers to the continuation of maceration after the completion of AF, during which the wine remains in contact with grape solids for an additional period of time before pressing [71,93]. PostFEM occurs in a fully hydroalcoholic medium, resulting in distinct extraction dynamics processes (Figure 1). The extraction is driven less by active mixing and more by diffusion processes and concentration gradients between the solid matrix and the surrounding wine, while cell structures become increasingly permeable due to the elevated temperatures during prior AF [7,16].

The duration of extended maceration is highly variable, ranging from several days to several weeks, even months, depending among others on grape variety, ripeness, and desired wine style [71,74,135,136]. In practice, short extensions of a few days are often used to refine phenolic balance, while longer macerations (e.g., 2–4 weeks or more) are applied to produce wines with increased structure, aging potential, and tannin complexity. The total duration of contact between grape solids and the liquid phase—deliberately extended during PostFEM—is likely the most critical factor in defining the phenolic profile and sensory properties of the resulting wine [16,137].

Compared with active AF, wine during PostFEM loses much of its natural protection, making the process more sensitive and demanding careful control. The loss of fermentative CO_2_ production increases oxygen exposure and alters redox conditions and can consequently influence phenolic stability, oxidation reactions, and microbial activity [7,70,136,138]. At the same time, the cap becomes less dynamic, which leads to a lower need for intensive cap management.

Within PostFEM conditions, temperature can be managed according to two different strategies. The first consists of allowing the temperature to decline naturally once AF ends, resulting in lower maceration temperatures than during active fermentation. Alternatively, some winemakers maintain moderate to warm temperatures (28–30 °C) to sustain extraction and limit microbial instability. To compensate for the loss of naturally produced CO_2_, tanks are often topped with CO_2_.

While extended maceration can enhance wine structure, it may also increase the risk of excessive tannin extraction, oxidation, and microbial spoilage [16,56,73,74,136]. These changes may lead to undesirable sensory attributes, particularly excessive bitterness and astringency, while oxidation and microbial activity can further compromise color attractiveness and aroma integrity [7,16,56,71]. However, warm PostFEM has also been reported to soften astringency and enhance sweetness perception in Bordeaux red wines [139]. The final outcome is likely influenced by grape variety, grape quality and maturity, as well as the extraction protocol employed.

Another aspect to consider, particularly in red winemaking, is that the altered chemical and microbiological environment during PostFEM may influence the progression of malolactic fermentation. In practice, this is often addressed by initiating malolactic fermentation during primary fermentation, allowing it to be completed by the end of the extended maceration period [138].

Nevertheless, despite certain risks and some potentially negative sensory outcomes, PostFEM is often applied to high-quality grapes and remains a popular practice among winemakers seeking to enhance wine structure and aging potential [56]. When carefully managed and adapted to the characteristics of the grapes, PostFEM benefits may outweigh the associated risks.

#### 2.5.1. Influence on Phenolic Compounds and Related Sensory Characteristics

Across numerous studies, PostFEM consistently emerges as a key driver of increased phenolic extraction, particularly of tannins [56,71,82,93,102,135,137,140,141,142,143,144,145,146].

In regard to **anthocyanins** and color-related parameters, PostFEM is generally associated with progressive changes in color density and hue as anthocyanins shift from monomeric and copigmented forms toward more stable polymeric and non-bleachable pigments [16,50]. Copigmentation initially enhances color intensity, but these non-covalent complexes gradually decline as anthocyanins participate in slower polymerization reactions with tannins, contributing to pyranoanthocyanin formation and greater color stability during maturation [50,118,147].

Such changes in anthocyanin composition and pigment profile have been documented in a series of studies on Merlot, Cabernet Sauvignon, Pinot noir, and Zinfandel wines [56,71,93,102,148]. Comparable trends, often accompanied by a shift toward a more evolved or browner hue, have also been reported in Tempranillo, Plavac mali, Babič, and Monastrell wines [137,140,144,149], together indicating that these changes represent a common outcome of prolonged skin contact across diverse red wine varieties.

The decline in anthocyanin concentration during PostFEM has been attributed partly to oxidative degradation and adsorption onto fermentation solids. However, their incorporation into oligomeric and polymeric pigments is considered the primary mechanism responsible for depletion of the monomeric pool [16,70,118,150].

**Flavan-3-ols** and **proanthocyanidins** are most clearly and consistently impacted by extended maceration. Numerous studies across various red varieties have shown that PostFEM increases the concentrations of these compounds, with many also corroborating a gradual shift in tannin origin over time [56,71,82,93,102,137,140,144,146,149,151].

The pattern observed across experimental studies is further supported by evidence from model wine systems. Extended maceration after AF was reported to promote the extraction of HMW seed tannins, whereas monomeric and LMW fractions were extracted predominantly at earlier stages [142]. Similar conclusions were reached in another study for flavan-3-ol extraction from seeds, which peaked after approximately 2–3 weeks of maceration [90]. Under reported experimental conditions, seed-derived compounds accounted for approximately 40–90% of total flavan-3-ol content, with their contribution increasing at higher alcohol levels [90]. Maceration duration influenced both flavan-3-ol concentration and composition, with gallocatechin identified as the major skin-derived flavanol and catechin as the predominant seed-derived flavanol [90]. Importantly, both model observations were subsequently re-evaluated in commercial red wine production.

Regarding the behavior of individual monomeric units, Teran wines produced with two different PostFEM durations showed significantly higher concentrations of procyanidin B1, (+)-catechin, procyanidin B2, (−)-epicatechin, and procyanidin B3 in the longer treatment (21 days PostFEM) compared to the shorter treatment (10 days) and control, supporting the importance of maceration duration [152].

Given the central role of tannins and anthocyanins in the PostFEM-related fate of phenolics, relatively few studies have focused on other phenolic classes. **Flavonols** are rapidly extracted during AF and generally decline after 8–9 days [16], suggesting limited effects of extended maceration. However, higher concentrations of kaempferol-3-glucoside were reported after 21 days compared with 10-day PostFEM treatments in Teran, suggesting that prolonged maceration may still affect its content [152]. The same study also documented slight increases in total **HBAs** and **HCAs** in the longer treatment, with most individual representatives showing similar upward trends, except for caffeic acid, which slightly decreased [152].

In addition, molecules with sweet taste extracted from grape seeds have been recently identified, such as epi-dihydrophaseic acid-3′-O-β-glucopyranoside and flavanonol astilbin. Nevertheless, the concentrations of these molecules remain unchanged during PostFEM [139,153].

Collectively, sensory studies confirm that the phenolic changes occurring during PostFEM are reflected in perceptible modifications of visual and mouthfeel attributes, frequently associated with altered color perception and changes in tannin perception, astringency, bitterness, and overall palate structure. However, the relationship between prolonged phenolic extraction and sensory perception does not appear to be straightforward, particularly with respect to astringency and tannin quality [71,135,142,143,144,152].

One possible explanation for these observations is that prolonged skin and seed contact may also promote the extraction of polysaccharides, which can interact with phenolic compounds and modulate wine mouthfeel [16,154]. In parallel, yeast autolysis continues during PostFEM, leading to the release of mannoproteins and other yeast-derived metabolites into the wine. Mannoprotein HSP12 released during yeast autolysis has been shown to contribute to sweetness perception in both red and white wines, while expression of the corresponding gene is particularly enhanced at temperatures above 30 °C [155,156]. Consistent with these findings, warm PostFEM was reported to soften astringency and increase sweetness perception in Bordeaux red wines [139]. More recently, N6-succinyladenosine was identified as another yeast-derived metabolite released during autolysis that may contribute to increased sweetness perception during PostFEM [157].

#### 2.5.2. Influence on the Aroma Profile: Chemical and Sensory Perspectives

The aroma-related consequences of PostFEM remain less clearly established than its phenolic effects, reflecting both the complexity of aroma formation and the comparatively limited number of available studies.

PostFEM of Aglianico di Taurasi wines for up to 90 days resulted in increased total ester concentration from 18.20 mg/L on day 8 (AF) to 52.57 mg/L on day 90 [158]. However, temporal trends were nonlinear and inconsistent with considerable variability among individual esters. Nevertheless, some of these results suggest that volatile composition may continue to evolve during prolonged post-fermentative skin contact long after AF has ended [158]. Notably, wines subjected to the longest PostFEM treatment exhibited the highest odor and taste complexity without developing off-odors or off-flavors [158].

A possible explanation for such continued aroma evolution may be found in the observations from Merlot wines subjected to PostFEM for up to 8 weeks, where 15 identified volatile compounds were significantly correlated with maceration length [135]. Yeast-derived volatile alcohols generally increased with extended maceration, except for phenethyl alcohol, while among terpenes α-terpinene, linalool, and nerolidol decreased and β-citronellol increased. The authors suggested that continued yeast metabolism after AF may have contributed to these trends during the post-fermentative period prior to pressing and potassium bisulfite addition [135].

A more recent study on Monastrell showed that extended maceration (146 days) generally reduced both the number and total relative abundance of volatile organic compounds (VOCs) [143]. Alcohols, terpenes, and sulfur compounds decreased in PostFEM compared to control, although selected compounds such as 2-ethyl-1-hexanol, phenylethyl alcohol, and ethyl decanoate increased significantly. In contrast, the overall relative abundance of esters and ketones was not significantly affected [143]. Nevertheless, these compositional changes were accompanied by lower sensory scores for aroma, fruity and floral attributes, while vegetal aroma tended to increase, suggesting that prolonged maceration may alter aroma balance in complex ways [143].

Another study investigating young Teran wines produced by different maceration strategies, including a 21-day PostFEM treatment, reported the most pronounced VOC changes associated with the longest maceration regime. Compared with shorter treatments, these wines exhibited lower concentrations of monoterpenes, alcohols, fatty acids, acetate esters, and ethyl esters, indicating a reduction in compounds commonly associated with fresh and fruity aroma expression [106]. In contrast, wines subjected to the longest maceration treatment exhibited the highest concentration of β-damascenone, together with higher 4-vinylguaiacol and lower 4-ethylphenol contents.

These compositional changes were accompanied by enhanced sensory complexity and more pronounced dried-fruit notes, likely linked to elevated β-damascenone concentrations [106]. Interestingly, a separate study reported that PostFEM enhanced the scores for varietal typicity of Teran [152].

Overall, while some earlier studies reported accumulation of selected alcohols, esters, or matured, tertiary aroma compounds during prolonged skin/seeds contact, more recent investigations point toward reductions in terpene- and ester-associated freshness, accompanied by enrichment of selected norisoprenoids and other volatile phenolic compounds. Collectively, these studies suggest that PostFEM may extend aroma development beyond the completion of AF, often leading to notable changes in wine volatile composition; however, in a cultivar- and process-dependent manner. Despite this variability, the available evidence indicates a general tendency toward a gradual shift from fresh, ester-driven aroma expression toward more mature and compositionally complex aroma profiles.

Future research should upgrade the view of PostFEM as a process driven primarily by continued phenolic extraction. Increasing evidence suggests that polysaccharides, flavanonols, yeast autolysis products, matrix-related interactions, temperature control and other concurrent chemical and biological changes may play important roles in shaping broader wine composition and sensory properties during prolonged skin contact. However, these factors remain less studied than phenolic extraction itself. Greater attention to these factors and processes may help explain the contrasting sensory outcomes reported among studies and provide a more complete understanding of the mechanisms governing wine evolution during PostFEM.

## 3. Technological Refinements and Advanced Modifications of Conventional Maceration Techniques

In addition to conventional, traditionally used maceration approaches, a range of technological refinements and advanced modifications have been developed to improve extraction efficiency, reduce time for the wine composition modulation, and expand stylistic possibilities in red winemaking.

Table 3 provides a condensed comparison of modified traditional maceration techniques, while further details on their extraction mechanisms, impacts on wine composition, and available research evidence are discussed in the following sub-sections.

### 3.1. Cryoextraction

Cryoextraction (CryoE) involves freezing of grapes or pomace to sub-zero temperatures prior to pressing and represents a more temperature-extreme pre-fermentative treatment than PreFCM, aimed to enhance the extraction of desirable compounds. Although often grouped within PreFCM, CryoE can be considered a modification of PreFCM or even a distinct procedure because ice crystal formation induces additional cell disruption mechanisms absent under typical PreFCM conditions. Ice crystals rupture pectocellulosic cell walls, damage grape skins, add air spaces in the cellular tissue, and thereby enhance the release of aroma and phenolic compounds from grape berries [17,159].

In practice, freezing can be applied using different approaches. In CryoE, grapes are frozen before pressing, whereas in supraextraction, freezing is combined with a gradual thawing phase prior to pressing [17,49]. It should be noted that both techniques require the grapes to be at least partially defrosted before pressing; however, the main difference is in the duration of the delay between thawing and pressing, rather than the freezing step itself [49]. It also needs to be emphasized that the terms cold maceration, cryomaceration, cryoextraction, and supraextraction are not clearly defined or consistently used in the literature. For example, treatments at approx. 5 °C have been described as cold maceration [22,57] or cryomaceration [79,160]; similarly, treatments at −20 °C have been referred to as cryomaceration [161], cryogenic maceration [162], and cryoextraction [163].

For freezing-based CryoE applications, the temperatures reported generally range from 0 °C to −20 °C, depending on the available technical possibilities, although values between −4 °C and −10 °C are most commonly used [17,159,164]. Low temperatures help prevent yeast activity and the onset of AF, while also limiting enzymatic oxidation and the loss of volatile aroma compounds [17,159,165]. In supraextraction, temperatures may be lowered to around −4 °C and subsequently allowed to rise again to approximately 10 °C before pressing, as originally explored to replace crushing in white wine vinification [17].

CryoE equipment, enabling rapid cooling of crushed grapes by using cryogenic gases, most commonly liquid carbon dioxide (CO_2_), is already commercially available. In these systems, a controlled amount of liquid CO_2_ is injected directly in-line into the grape mash stream to achieve a pre-set target temperature. The amount of the CO_2_ required depends on both the mass of the material being cooled and the desired final temperature, with consumption increasing as the temperature differential becomes greater [159].

#### 3.1.1. Influence on Phenolic Compounds and Related Sensory Characteristics

Limited evidence suggests that CryoE can modify the phenolic composition of red wines, although the reported trends are not always consistent across studies.

Pre-fermentative maceration of Sangiovese must at −5 °C, 0 °C, and +5 °C was investigated using dry ice and liquid nitrogen (the latter only for 0 °C and +5 °C), alongside untreated controls [57]. In dry ice-treated grapes, total phenolics extraction was best promoted at the lowest maceration temperature, with anthocyanin–tannin condensation (dTA) showing a similar trend. When TA was expressed as a percentage of total anthocyanins (TA%), differences between batches were negligible, indicating that higher dTA resulted from increased extraction rather than changes in anthocyanin distribution. Under comparable conditions (T), liquid nitrogen was more effective than dry ice [57]. Sensory evaluation further revealed temperature-dependent differences, with wines processed at −5 °C receiving higher scores for color intensity, hue and tannin perception than both the control and 0 °C treatments [57].

In Negroamaro, CryoE (24 h; 0 °C; dry ice) increased anthocyanin concentration but reduced TPC compared to traditional vinification, suggesting a more selective extraction of skin pigments [164]. In another study applying dry ice to briefly freeze destemmed berries (−1 to −4 °C) modified phenolic composition in both Pinot noir and Cabernet Sauvignon [166]. In Pinot noir, the treatment enhanced red pigment coloration and increased delphinidin-3-acetylglucoside concentration, whereas Cabernet Sauvignon exhibited an increase in tannin content and altered color hue. In sensory analyses, Pinot noir wines were perceived as more intensely colored. Cabernet Sauvignon wines, on the other hand, exhibited greater volume and reduced dryness compared with the control [166].

These findings together support that CryoE-related phenolic outcomes in red wines are influenced by grape variety and treatment conditions.

#### 3.1.2. Influence on the Aroma Profile: Chemical and Sensory Perspectives

Studies on volatile composition as affected by CryoE are more frequent in white wines; however, in Sangiovese, CryoE, followed by AF at two temperatures (20, 30 °C), was compared with control and PreFCM treatments. Distinct volatile profiles were observed in the case of some individual esters and higher alcohols [167]. At 20 °C, CryoE wines were the only samples containing detectable hexyl acetate and showing higher 1-hexanol together with lower 1-butanol concentrations. At 30 °C, CryoE wines exhibited the highest levels of ethyl decanoate and 4-ethyl-phenol, although the latter remained below its sensory threshold [167]. In addition, five vinification treatments were compared in Busuioacă de Bohotin wines, with classical control and CryoE among them. CryoE caused changes in several volatile compounds, including some alcohols, esters, and terpenoids [161]. However, the absence of statistical analysis limits the strength of the conclusions that can be drawn from these data. Sensory analyses further revealed temperature-dependent changes in Sangiovese. Wines processed at −5 °C received lower scores for flavor complexity and balance compared with both the control and 0 °C treatments, while flavor intensity remained similar among treatments [57]. Furthermore, enhanced notes of soft fruits and licorice in CryoE Negroamaro wines and increased cinnamon notes in Pinot noir wines were also observed [164,166].

Overall, it is important to keep in mind the substantial variation in terminology used for specific temperature treatments (see above). This indicates that, in both practical and experimental contexts, the boundary between different cold-temperature approaches remains unclear. Consequently, the available findings should be interpreted cautiously and always in relation to the specific vinification conditions under which the wines were produced. In addition, studies on red wines remain very limited, highlighting the need for further investigation using more integrated and standardized research approaches together with the advantages of advanced analytical techniques and focused sensory analyses.

### 3.2. Accentuated Cut Edges

Accentuated Cut Edges (ACE) is a recent extraction technique in which grape skins are cut or shredded into small fragments (approximately 10% of their original size) without damaging the seeds [125,168].

The goal is to facilitate the release of phenolic compounds from the skins into the must by increasing the total surface area and the number of open or accessible skin edges [169]. The ACE step is applied after destemming and crushing, thus can be classified as a pre-fermentative technique. Equipment capable of performing ACE is, for instance, the Della Toffola maceration accelerator [168]. ACE procedure typically allows earlier pressing, which shortens the maceration period and reduces the need for cap management, ultimately saving valuable cellar time, improving fermentation tank efficiency, and lowering operational costs [168]. Consequently, it represents an interesting candidate for practical winery application; however, current knowledge regarding the procedure, its possible variations, and their influence on the chemical and sensory properties of wine remains insufficient.

#### 3.2.1. Influence on Phenolic Compounds and Related Sensory Characteristics

The ACE technique has been generally shown to enhance phenolic extraction in red wines, though the magnitude of this effect depends, among others, on application timing, grape matrix, and accompanying treatments.

In a study on Pinot noir wines, originating from grapes processed by ACE, the highest proportion of red color, non-bleachable pigments and tannins, compared to other treatments and control, was observed at six months of bottle aging, with ACE further reported to be considerably more effective than submerged cap vinification for enhancing these parameters. However, on the palate, bitterness and astringency were also substantially increased in ACE wines compared to control [169].

Regarding the timing of application, in Marquette interspecific hybrid grapes, ACE applied at crushing increased color intensity, non-anthocyanin monomeric compounds, and total iron-reactive phenolics compared to control as well as compared to ACE applied 24 h before pressing, with these differences remaining stable after five months of aging [170]. The findings indicate that application timing may play an important role in procedure efficiency for certain phenolic groups. However, in the same study, condensed tannin concentrations remained below detection limits in all Marquette samples, suggesting that ACE either did not aid tannin extraction or that further interactions occurred with disrupted cell wall material [170]. In another study on Marquette wines, the combination of ACE with macerating enzymes was shown to be particularly effective, improving monomeric phenolics by 20% and tannins by 21% after nine months of aging, while both treatments when applied separately had little impact [171].

In Shiraz wines, ACE significantly enhanced total tannin and phenolic concentrations without visually affecting color. In parallel, the same authors observed that polysaccharide concentration was influenced more by maceration time than by the ACE technique itself [172]. Contrary to findings in Pinot noir [169], two related studies on Syrah reported no significant differences in bitterness and astringency [168,172]. However, these observations were based on a consumer preference study rather than evaluation by a trained sensory panel.

The level of skin fragmentation via different lengths of ACE procedure also matters. In Shiraz musts diluted with water to reduce alcohol levels in response to climate change-driven increases in grape sugar concentrations, long ACE treatment (40 s, compared to 20 and 10 s) restored total phenolics and tannins to values similar to the non-diluted control, but not free anthocyanins, suggesting that increased skin fragmentation may compensate for the dilution effect on some, but not all, phenolic groups [173].

#### 3.2.2. Influence on the Aroma Profile: Chemical and Sensory Perspectives

There is little evidence on the ACE effect on wine aroma composition; however, the impact of ACE on wine volatile profile appears to be mainly mediated by skin contact time and the interaction with other winemaking parameters.

A study on Pinot noir revealed that ACE treatments increased ethyl butanoate concentrations, whereas ethyl 2- and 3-methylbutanoate as well as ethyl 2-methylpropanoate generally decreased as compared to the untreated control. Ethyl propanoate and ethyl acetate were not affected by ACE itself, but decreased when combined with submerged cap treatment. Acetates (2- and 3-methylbutyl acetate) and butanol were also found to increase in ACE wines compared to control wines [169]. Sensory evaluation of the experimental wines after 6 months generally revealed lower average scores for red fruits and confectionary-like odor, but higher for dark fruits compared to control [169].

In Shiraz wines, the influence of ACE on volatile composition in combination with skin contact time (3 or 6 days) was evaluated, with particular focus on varietal thiols. ACE only marginally increased thiol precursor concentrations in must, but was later associated with significantly higher consumption of glutathionylated precursors of 3SH during AF. While ACE itself did not significantly affect 3-SH or 3-SHA formation, shorter skin contact (3 days) tended to increase 3-SH concentrations compared to 6-day maceration. Regarding other volatile compounds, ethyl ester formation was generally higher in control, whereas the ACE treatment combined with short skin contact showed potential to enhance higher alcohols, fatty acids, and certain terpenes such as α-terpineol. Consumer-based sensory evaluation highlighted stronger vanilla and floral/perfume/musk notes, particularly after shorter ACE treatments [168]. The same authors also briefly assessed the volatile profiles of Sauvignon Blanc and Pinot noir wines produced at commercial scale with ACE implementation, with results indicating that the effects of the treatment may strongly depend on grape variety [168,172].

Overall, although ACE appears promising, particularly for enhancing phenolic extraction and modulating wine style, the available evidence remains very limited and often arises from the same or closely related experimental designs. Further research is therefore needed to improve the understanding and optimize processing conditions, evaluate combinations with other vinification steps, and better clarify its chemical and sensory consequences, particularly through integrated studies using trained sensory panels combined with well-controlled winemaking conditions and with a broader range of cultivars included.

### 3.3. Thermovinification

**Figure 2:**
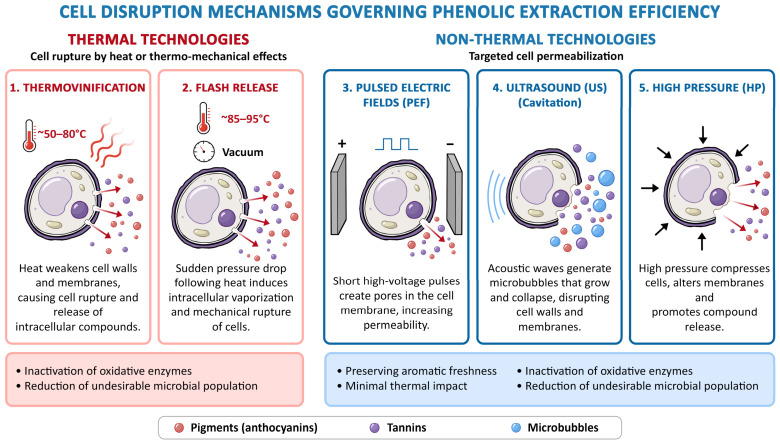
Cell Disruption Mechanisms: Thermal vs. Non-Thermal Technologies.

Thermovinification (TV) is a thermal pre-fermentative technique primarily developed to enhance, and particularly accelerate, the extraction of phenolic compounds, especially anthocyanins, into the must or wine by promoting the disruption of grape cell walls (Figure 2). The process involves heating crushed grapes or must, most commonly in tube-in-tube heat exchangers, to temperatures ranging from 50 to 80 °C while maintaining contact between the liquid and solid grape components. Importantly, temperatures should generally not exceed 85 °C, because excessive heating may intensify undesirable effects on wine composition and sensory quality [17,74,79,174].

Heating may last from a few minutes to several tens of minutes, whereas treatments extending for several hours are sometimes referred to as “hot vinification” or pre-fermentation hot maceration (PreFHM) [74,174,175]. A variation in PreFHM developed in Germany, known as KZHE (short-time high-temperature treatment with warm maceration), involves heating crushed grapes to approximately 85 °C for 2 min, followed by warm maceration at around 45 °C for 6–10 h before fermentation without solids [174,175].

After TV heating, the pomace is pressed before fermentation, which distinguishes TV from conventional red winemaking. The must is then rapidly cooled, typically to around 35–40 °C, to allow yeast inoculation and AF. Besides creating suitable fermentation conditions, this cooling step may further contribute to grape skin disruption and facilitate extraction. In many cases, the must is clarified before fermentation, which then proceeds mainly in the liquid phase, unlike “hot vinification,” where fermentation can continue in contact with solids [118,122,175].

Besides promoting rapid phenolic extraction, TV also provides several technological advantages linked to the use of elevated temperatures, including inactivation of oxidative enzymes (e.g., PPO) involved in browning reactions, and reduction in undesirable microorganisms. Molds, non-*Saccharomyces* yeasts, acetic acid bacteria, and lactic acid bacteria are highly sensitive to temperatures employed during TV, and their populations are markedly reduced or killed, with the effect becoming more pronounced at higher temperatures [74,79,125].

However, these advantages may also be accompanied by certain decreases in wine quality. Exposure to very high temperatures can lead to the loss of volatile aroma compounds on one side and the formation of undesirable odors, resulting from thermal degradation, on the other side [176]. Interestingly, increasing temperature was associated with lower concentrations of 2-methyl-3-furanthiol, a compound potentially linked to the Maillard reaction [177]. Although TV generally promotes phenolic extraction, these extracted compounds may later exhibit lower colloidal stability, which can potentially enhance the precipitation of anthocyanins and tannins during storage. TV may also alter wine pH, thus further implicating chemical stability and sensory balance of final TV-treated wines [74].

#### 3.3.1. Influence on Phenolic Compounds and Related Sensory Characteristics

Despite the general associations of TV techniques with enhanced phenolic extraction and wine color characteristics, research evidence does not always support these benefits. Overall, relatively limited experimental work often reports increased phenolic extraction and color expression, but its effects on individual phenolic groups are strongly dependent on grape variety, vintage, heating time and intensity, and the vinification protocol applied. The response of anthocyanins to TV treatments appears particularly variable, with some studies reporting elevated levels, while others observed either partial degradation or no clear content improvement despite visually enhanced color intensity. In a study on Grenache, Carignan and Fer TV-treated wines, it has been observed that solid-phase fermentations (S) after heating tended toward higher anthocyanin concentrations, whereas liquid-phase fermentations (L) more often resulted in their decrease, with outcomes clearly influenced by both cultivar and vintage [176]. Total phenolic index (TPI) appeared even more cultivar-dependent, with both increases and decreases reported, and no consistent trend as to whether S or L produced higher values [176]. In a following study performed on Carignan, TV temperature and duration only mildly influenced the extraction of phenolic compounds. Even though extended heating at 50 °C achieved phenolic levels similar to those obtained by short treatment at 75 °C, the higher temperature appeared to promote anthocyanin degradation [177]. Additional insight was provided by TV-treated Cabernet Sauvignon-based model musts (70 °C for 30 min) exhibiting increased color intensity of freshly fermented samples by about 60% compared to untreated control, while TP and flavonols increased by 32% and 97%, respectively [178]. In contrast, TA concentrations at the end of AF did not differ significantly from those in control, suggesting that color enhancement was not directly linked to anthocyanin accumulation [178]. Similarly, regarding TP, increased concentrations were reported in TV-treated Merlot, Cabernet Sauvignon, Pinot noir, and Prokupac wines [179], irrespective of the heating protocol applied (60 °C for 1 h or 80 °C for 3 min) [179]. More recently, TV was compared with enzyme-assisted and traditional maceration in Babica wines. TV wines showed the lowest hue values, but the highest color intensity together with nearly double TPC and total flavonoids compared to other treatments. However, TV wines also contained the lowest concentrations of tannins and anthocyanins, despite an initial increase in the latter [180]. Partly in line with the results of chemical analyses, sensory evaluation revealed TV-related enhancement of color perception together with stronger astringency and slightly improved body fullness [180].

Regarding the effect of temperature, TV treatments on Syrah at 55, 65, and 75 °C were evaluated compared to traditional 7-day macerated wines [181]. Heating generally intensified wine color and notably increased trans-caftaric acid content while also negatively affecting other phenolic acids. However, higher temperatures, especially 75 °C, promoted anthocyanin degradation while simultaneously enhancing some flavonols and flavan-3-ols. Overall, in their study conditions, TV at 65 °C seems to provide the best balance between color stability, unwanted anthocyanin loss, and related wine quality improvement [181].

#### 3.3.2. Influence on the Aroma Profile: Chemical and Sensory Perspectives

Several studies indicated that TV can also modify the volatile composition of red wines. Pre-fermentation heating caused a decrease in the levels of several volatiles, including some norisoprenoids, terpenols, and volatile phenols, while also suggesting thermal degradation and the appearance of some heat-related aroma [176]. Regarding temperature and treatment duration, it was indicated that heating temperature had a stronger influence on Carignan wine aroma composition than treatment duration, although the magnitude of these effects significantly depended on vintage. Several varietal aroma compounds were altered at higher temperatures, with treatments processed at lower temperature (50 °C) generally retaining higher concentrations of terpenols and norisoprenoids, including β-citronellol, geraniol and β-damascenone [177]. In the case of Marselan, TV-assisted winemaking was associated with higher ester concentrations than the untreated control, probably due to must enrichment in organic nitrogen and the occurrence of alcoholic fermentation in the liquid phase [182]. Sensorially, these experimental wines exhibited more pronounced fruity notes, but less expressed spicy, overripe and vegetal notes compared to wines produced by the untreated control.

Overall, TV appears to be a useful, however highly context-dependent technique, with both advantages and limitations that should be carefully considered before implementation (Table S1). TV may be particularly valuable for processing infected or otherwise compromised grapes as well as during abundant vintages, when too fast grape intake and limited cellar capacity become important practical constraints. Consequently, it deserves further research attention, particularly in terms of optimizing processing conditions that would enhance benefits and limit drawbacks, together with a better understanding of the key factors affecting a wider range of phenolic, volatile and other compounds and their combined impact on final wine composition and quality over the long term.

Although TV was introduced already in the 1960s, it has never become widely established in premium wine production. This has also led to ongoing efforts to refine processing conditions building on TV benefits, ultimately leading to the development of related modifications such as flash release.

### 3.4. Flash Release

Flash release (FR), often referred to as flash détente or flash expansion, is a modification of thermovinification in which grapes are exposed to even higher temperatures while simultaneously subjected to a strong vacuum. This dual action leads to the rapid evaporation of water, the rupture of grape cell walls, and consequently, an improved extraction of mainly phenolic compounds (Figure 2) [125,174,183].

More specifically, FR is a continuous procedure involving brief heating (typically for 5–15 min) to 85–95 °C, usually by direct steam injection, followed by near-instantaneous cooling to 30–35 °C in a vacuum or expansion chamber operated at 20–60 hPa [125,174,184,185]. Oxygen exclusion from the heating atmosphere helps to avoid overheating and reactions such as Maillard browning. However, some systems only reach 80–85 °C rather than the preferred temperature closer to 90 °C, while extraction efficiency decreases markedly below 70–75 °C [83,186].

The process requires specialized equipment, including a heat exchanger, a vacuum chamber operated under strong negative pressure, and a boiler for rapid steam generation [174], together representing a substantial technological investment accompanied by considerable operational costs [175].

The characteristics of FR wines can be further modulated by conducting AF either in the liquid phase only or in the presence of grape solids for varying periods of time [174]. It has also been reported that the yeast lag phase prior to fermentation may be slightly shorter in flash release-treated grape pomace, likely because the disruption of grape tissues increases the release and availability of yeast nutrients [174,183].

The process was developed and patented in 1993 by the French National Institute for Agricultural Research (INRA) in collaboration with Aurore d’eveloppement and Imeca-Della Toffola with the aim of providing an efficient method for rapidly increasing phenolic compound concentrations in both must and finished wines [187,188].

#### 3.4.1. Influence on Phenolic Compounds and Related Sensory Characteristics

Although limited, available studies generally support FR as a possible tool to enhance the extraction of phenolics from grape skins and sub-skin vacuoles; however, extraction yields among phenolic families and the persistence of these effects in final wines vary considerably.

Investigation of phenolic extraction during CF+M following FR must treatment (Grenache, Mourvèdre and Carignan) revealed that, compared to untreated controls, anthocyanins, flavonols, HCAs, catechins, and proanthocyanidins were substantially higher in FR musts before AF [189]. Following fermentation, flavonol, catechin, and proanthocyanidin concentrations further increased, whereas HCAs generally decreased. Anthocyanin concentrations remained slightly higher; however, the differences between control and FR wines became much smaller than those observed before AF [189].

To assess the effects of heating time and solids retention, FR for 6 min, FR for 6 min followed by fermentation in liquid phase, and FR for 15 min were compared in Grenache and tested against untreated control. FR with solids increased the total phenol index (TPI), with further increase after prolonged heat exposure. In contrast, wines fermented in the liquid phase showed substantially lower TPI and color intensity, highlighting the importance of skin contact for anthocyanin extraction and pigment formation [185]. Color intensity itself was initially only slightly affected by FR, but increased after longer heat exposure [185].

In Pinot noir, the results were less convincing. Although FR-treated musts initially showed higher TPC than controls, mainly due to markedly higher anthocyanin (approximately 10-fold) and phenolic acid content, these differences almost disappeared during fermentation and maturation. After 10 months in bottle, TPC in FR wines tended to be lower than in controls, while anthocyanin and phenolic acid concentrations did not differ significantly between treatments. Flavan-3-ols, however, remained largely unaffected throughout the whole trial period [190].

Fermentation temperature may further influence FR outcomes. In Cabernet Sauvignon produced from FR-treated musts fermented in the liquid phase at 16, 24 or 32 °C, higher temperatures reduced catechin, epicatechin and malvidin-3-glucoside concentrations, but increased pigmented polymers and non-bleachable pigments. No clear temperature effect on total phenolics or proanthocyanidins was observed [187].

Additional evidence from Cabernet Sauvignon indicated higher levels of condensed tannins at all polymerization degrees in FR wines, further showing greater color intensity and higher levels of total monomeric anthocyanins, malvidin derivatives and flavonols, while reducing phenolic acids. Similar behavior was also observed for Marselan FR wines [188]. Accordingly, in terms of mouthfeel, FR wines exhibited greater astringency and bitterness [188], while enhanced extraction of polysaccharides and proanthocyanidins may further influence wine body and astringency [187].

Overall, reported effects of FR on phenolics are too variable and the available evidence too limited to establish reliable trends across studies.

#### 3.4.2. Influence on the Aroma Profile: Chemical and Sensory Perspectives

Research on FR has only recently begun to focus on aroma, and related studies are still scarce. In Cabernet Sauvignon, lower fermentation temperature and reduced solids led to increased ester concentrations, whereas higher temperatures and greater solids content promoted fusel alcohols and linalool formation [191]. Differences between liquid fermentation and fermenting with solids were also notable sensorially, with more pronounced red fruit and confectionery notes in the former and a more pronounced fruit character in the latter. FD wines also exhibited weaker green and savory attributes than control [191].

In Marselan and Cabernet Sauvignon wines, FR treatments generally resulted in increased esters, lactones, and furanone concentrations, while C6 alcohols and C13-norisoprenoid levels tended to decrease [188]. Compared with traditional maceration, FR wines also showed more pronounced fruity, particularly fresh-fruit, characters, together with sweeter sensory notes. Consistent with earlier observations on Cabernet Sauvignon [191], both Marselan and Cabernet Sauvignon exhibited weaker green and floral notes [188].

FR effects on wine phenolics, volatile profile and sensory properties are highly variable among studies and appear to depend on numerous factors that are still not sufficiently understood. Moreover, the persistence of many of these effects throughout maturation is unclear; thus, justifying the high technological investment and operational cost associated with FR remains difficult. Future research should focus on improved understanding of the long-term behavior of FR-related effects and optimizing processing conditions across different winemaking strategies and their combinations.

## 4. Maceration by Emerging and Non-Thermal Technologies

Emerging, non-thermal technologies have been recently introduced for food and beverage processing as alternatives to traditional thermal treatments (Figure 2). Thanks to scientific research, some of them have been successfully applied in winemaking processes, especially with regard to the inactivation of spoilage microorganisms and the extraction of polyphenols and other bioactive compounds. To date, ultrasound, pulsed electric fields (PEF) and high-pressure processing have been authorized by the International Organization of Vine and Wine (OIV) for processing grapes and must. On the other hand, microwave-assisted extraction and ohmic heating are very interesting and promising tools for improving the extraction of polyphenols and preserving their antioxidant properties due to the lower impact of thermal damage, without compromising the chemical and sensory quality of the treated products. Nevertheless, these two latter techniques have not been authorized yet for their application in winemaking.

A direct comparison between the main advantages, OIV status and impact on wine quality by the different emerging technologies is reported in Table 4, while operating conditions applied, grape variety and the main results obtained for each technology are schematized in more detail in Table S2 (Supplementary Materials).

The recent advances about the use of ultrasound, pulsed electric fields, high-pressure processing, microwaves and ohmic heating as tools for accelerating extraction of polyphenols and reducing the maceration time will be illustrated in the following section.

Despite the interesting results offered by these emerging technologies for reducing the maceration time while maintaining or even improving wine quality, more efforts are necessary to scale up the operating conditions and, in general, to optimize the process. Furthermore, some of them are already authorized for their application in winemaking and several installations are already available (i.e., for PEF, US), but the current high cost of plants and equipment prevents them from widening in the enology field.

### 4.1. Ultrasound

#### 4.1.1. Mechanism of Action

Among emerging, non-thermal technologies, ultrasound (US) has been authorized by OIV for processing must, in order to stimulate extraction of grape compounds and reduce maceration time [192]. The enhancement of phenolic extraction from grape cells is mainly a consequence of cavitation phenomena, reflected in the generation of microbubbles by the acoustic waves applied and their further collapse. The implosion of microbubbles also determines the formation of local hot spots, with a consequent increase in temperature and pressure, causing cell injury and disruption [193]. In these conditions, the cleavage of other molecules, mainly water, also occurs, thus determining the formation of free radicals and other reactive oxygen species that further cause cell damage [194].

#### 4.1.2. Impact on Wine Quality

The application of this technology in red winemaking allows consistent reduction in the time needed for maceration, thanks to the higher extraction of phenolic compounds, anthocyanins, tannins and stilbenes, together with an increased concentration of varietal aroma compounds, compared to conventional maceration techniques [195,196].

The application of frequency, generally ranging from 28 to 40 kHz, increases the concentration of anthocyanins and total tannins from two to ten times compared to non-sonicated must, with a significant increase in color intensity and stability even after AF and aging [195]. The addition of pectolytic enzymes on crushed grapes, followed by US treatment, seems to amplify the extraction rate of phenolics, probably due to a synergistic effect of these two techniques; this combined treatment has consequentially determined an increase in the content of tannins and anthocyanins of 7% and 16%, respectively, compared to enzyme addition alone [197].

Regardless of processing parameters, the efficacy of US in enhancing the extraction rate and shortening processing time allows obtaining equal or even higher concentration not only of polyphenols [195], but also of sugars, yeast assimilable nitrogen and grape polysaccharides, compared to both cold and warm traditional maceration techniques carried out for several days [196,198,199,200]. This might also significantly impact the overall winemaking process, in terms of time and costs.

Different prototypes or pilot-scale plants are available, offering the possibility to manage processing parameters, namely frequency, power, time of treatment and temperature, with the aim to optimize grape processing and extraction, while preserving the chemical composition of grapes, must and wines [195,196,201]. In addition, commercial plants specifically designed for winemaking are available on the market, in different dimensions, working flows and power input.

Some studies also reported that prolonged maceration of ultrasound-treated grapes may lead to a decrease in phenolic compounds, mainly total tannins, probably due to a concomitant extraction of grape polysaccharides or adsorption phenomena on the solids fraction of grapes [195]. However, the extraction of polysaccharides may also be beneficial for improving wine color stability already during the first steps of the winemaking process, thus potentially increasing the quality of low- to medium-range wines too.

Treating grapes with US also determines the reduction in total yeasts, molds and bacteria up to 90% after 90 min of treatment at a frequency of 40 kHz, with a treatment temperature of around 25 °C, reaching a final microbial concentration of 100–200 CFU/mL [196]. By reducing spoilage microorganisms, these treatments may also decrease the need for sulfur dioxide addition, facilitate fermentation management by supporting the implantation and activity of starter yeasts, and contribute to improving wine quality and safety.

Besides higher color stability, red wines obtained by US-assisted maceration also show improved physicochemical properties even after aging, as well as a more acceptable sensory profile, thanks to the higher content of varietal aromas, mainly terpenes, C6-alcohols and norisoprenoids [202,203] and the formation of more stable pigments due to polymerization reactions [195,204].

Overall, US represents a powerful technology for enhancing the extraction of key compounds with lower thermal impact, and may consistently reduce the maceration time, consequently improving the overall quality and stability of red wines. Other possible applications might be linked to the extraction of aroma precursors, inactivation of oxidative enzymes and spoilage microorganisms, improvement of protein stability and lees processing. However, the technology has several limitations, including its applicability primarily to liquids or crushed grapes, the risk of thermal degradation if not properly applied, and the limited number of grape varieties investigated to date. Further studies are needed to update the knowledge needed to assure more consistent outcomes and to better standardize the process depending on grape varieties, ripeness and related vinification methods.

### 4.2. Pulsed Electric Fields

#### 4.2.1. Mechanism of Action

Pulsed Electric Fields (PEF) technology is based on the application of short pulses of high-voltage current to solid or liquid products positioned between two electrodes [205]. Typically, the electric field strength is between 0.1 and 80 kV/cm, with pulses from microseconds to milliseconds long [205,206]. When applied to food products, living tissues, microorganisms, or vegetal matrices, PEF provokes structural changes in cell membranes, creating pores and leading to a temporary increase in membrane permeability or to an irreversible cell breakdown [205,207,208,209,210].

The ability of PEF to cause the loss of membrane semipermeable properties, inducing leakage of intracellular content, was first investigated on microorganisms in the 1960s [211,212,213]. Between the 1970s and early 1990s, several authors contributed to clarifying the concept of “electroporation/electropermeabilization” [209,210,214,215,216], referring to the formation of hydrophilic pores in the cell membrane, caused by the dielectric polarization and re-orientation of the phospholipid bilayer that occurs when the electric field is applied. This phenomenon can be reversible or irreversible, depending on factors such as the intensity of the electric field or the number and duration of pulses [205,217]. Since the 1990s, PEF has been tested in several studies for improving the extraction of intracellular and bioactive components from vegetal matrices [218,219,220,221], including grapes.

#### 4.2.2. Impact on Wine Quality

The first reports on the application of PEF to improve maceration in red winemaking date back to 2008 [222]. In these papers, PEF-induced permeabilization of grape skin tissues was found to be an effective treatment compared to the controls for improving the extraction of phenolic compounds, reducing maceration time and accelerating wine processing, leading to increased wine color intensity, anthocyanin and total polyphenolic content [223,224,225]. In the last two decades, several other papers have been published regarding the optimization of PEF technology for improving and accelerating skin maceration, obtaining comparable results; Table S2 reports an overview of the effects described in the most relevant documents. In similar processing conditions (total specific energy of 11–22 kJ/kg), PEF was also able to increase the release of varietal aroma precursors from grapes, without enhancing the concentration of C6 volatile compounds [226,227].

Interestingly, the compositional differences between controls and PEF-processed samples found in young wines at the end of maceration/alcoholic fermentation remain relatively stable throughout the whole winemaking process, as well as during wine storage and aging [228,229,230].

The typical installation setup used for the PEF treatment of grapes consists of a high voltage DC generator, a bank of capacitors able to store great amounts of energy and discharge it instantaneously, an electrical switch, sensors used to measure the high-voltage and the current delivered to the chamber, an oscilloscope to monitor the form of the pulse wave, a treatment chamber where the electrodes are arranged in a collinear fashion along the pipe flow path, and a pump (e.g., single screw pump) suitable for moving grape mash [205].

In general, the advantages offered by PEF should be discussed not only in terms of efficiency, but also from a sustainability point of view. In fact, the possibility to significantly reduce maceration time reflects a substantial benefit in terms of energy savings, due to the reduced need for refrigeration to maintain the temperature of the fermenting mash. Moreover, in this regard, PEF processing of grapes can be carried out at room temperature, as it does not induce heating; consequently, temperature control during treatment is not required. López and colleagues found that the temperature of the samples never exceeded 30 °C in experiments with a total specific energy of 1.8 and 6.7 kJ/kg [222]. Comuzzo et al. reported that the temperature of the mash increased by less than 3 °C after PEF treatments at 2, 10 and 20 kJ/kg [228]. Puertolas and co-workers indicated a temperature increment due to the treatment lower than 2 °C, for a total specific energy of 3.67 kJ/kg [230]. Other papers report similar results [174,229], confirming that PEF processing is a low-impact and energy-saving technology that allows better preservation of grape quality, compared to other techniques used to accelerate the extraction of color and phenolic compounds from grapes (e.g., thermovinification).

Finally, unwanted effects reported in the literature for PEF treatments, such as the corrosion of the electrodes with the release of metal ions (iron, chromium and nickel) [231,232], generally appeared to have a low relevance in the conditions normally applied during grape and wine processing [228,233]. However, there are very few publications dealing with these aspects, and this issue should be further investigated in the future [234].

The treatment of grapes by PEF has been included in the OIV International Code of Oenological Practices in 2020 [235] and, since 2022, has been authorized for use at winery scale by European legislation [236].

### 4.3. High Pressure Processing (HPP)

#### 4.3.1. Mechanism of Action

High pressure treatments have been introduced in the food industry as an alternative to traditional pasteurization for the inactivation of spoilage and pathogenic microorganisms in beverages [237,238,239]. In this context, OIV has authorized this emerging, non-thermal technology for processing grapes or must with the main aim of reducing wild microorganisms’ population. HPP may be performed in discontinuous mode, such as high hydrostatic pressure (HHP), or in continuous processing mode, namely high pressure homogenization (HPH) or ultra-high pressure homogenization (UHPH) [240,241]. The effect on cell breakage—causing both microorganisms’ inactivation and an improvement of the extraction rate from grape cells—is mainly due to the mechanical damage of cell structures, walls and membranes. This damage is linked to the pressure applied to the food through a pressurized liquid—generally water—in the case of high hydrostatic pressure, or to the intense shear and impact forces when a fluid passes through a valve, in the case of high or ultra-high pressure homogenization [242].

Both these technologies offer different advantages; besides microbial inactivation, they also promote the extraction of polyphenols and aroma precursors, with a consequent reduction in maceration time. These technologies have demonstrated interesting results about the inactivation of oxidative enzymes, being a powerful tool for preserving browning and therefore applicable also in white winemaking. Nevertheless, some limitations may occur at the operating level. Ultra-high-pressure homogenization may be applied in continuous mode, thus reducing processing time, but the application on crushed grapes containing a solid part may determine a rapid clogging of the homogenizing valve. This problem might be overcome by using high hydrostatic pressure, which allows processing also uncrushed grapes. Some industrial plants are already operating for other food applications, i.e., for pasteurization of fruit juices, but the discontinuous mode may limit the quantity of grapes and must to be processed, consequentially prolonging the time and increasing the costs of the process.

#### 4.3.2. Impact on Quality by High Hydrostatic Pressure (HHP)

During high hydrostatic pressure treatment (HHP), pressures above 200 MPa, especially between 300 MPa and 400 MPa, are applied for a very short time (up to ten minutes). This condition seems to be necessary and sufficient to completely inactivate wild microorganisms, with a total reduction in native yeasts (<10 CFU/mL) and a residual bacterial load of about 100 CFU/mL [243,244]. This consequentially allows for reducing sulfur dioxide addition and faster implantation of yeasts used as fermentation starters, without compromising the overall wine quality and aroma profile [245]. Even though OIV has authorized the application of this non-thermal technology only for sterilizing grape must, a non-negligible effect of HHP on polyphenols extraction from blueberry pomace [246,247,248], grape pomace [249,250,251] or grape juice [252] was also assessed, especially when combined with enzyme addition [249,253]. However, the efficiency of the extraction yield and total content of phenolic compounds also depends on the pressure applied and the temperature reached during the treatment, which may determine a loss of these compounds [251,254]. Prolonging treatment time or pressures exceeding 600 MPa seem to determine a loss of total phenolic content and anthocyanins and a decrease in the antioxidant activity in HHP-treated must, while simultaneously reducing the wild microbial population [254]. Under winemaking conditions, HHP applied at 300 MPa for 2 min favored the extraction of both anthocyanins and organic acids, thus enhancing copigmentation rate, thermal stability and increasing antioxidant activities [255]. The content of anthocyanins and tannins significantly increased immediately after HHP treatment in Cabernet Sauvignon and Merlot grape must [256], possibly increasing during maceration. This enhanced release might possibly determine a greater total polyphenols content and higher color intensity in the resulting wines, probably due to the higher presence of anthocyanins in the acetylated forms [243,250]. The application of HHP on grapes before maceration also determined a higher content of methanol due to an enhanced hydrolysis of grape pectin caused by pressure. In addition, a greater concentration of higher alcohols (at 600 MPa) and esters (at 200 and 400 MPa) was observed in the resulting wines, contributing to a better aroma quality with a more intense fruity profile [243]. The greater preservation of polyphenols in the final wine may also be linked to the efficacy of HHP on inactivating oxidative enzymes, peroxidase (POD) and polyphenol oxidase (PPO), which, however, depends on processing parameters, mainly temperature and pressure [256,257,258,259].

#### 4.3.3. Impact on Wine Quality by Ultra High-Pressure Homogenization (UHPH)

Like HHP, UHPH has also been authorized by OIV for inactivating native microorganisms and for reducing sulfur dioxide addition, without compromising wine sensory quality [241]. By comparing high-pressure treatments in continuous and discontinuous mode in processing red sugar cane from different varieties, Mansor et al. suggested that HPH is more effective in inactivating microorganisms and polyphenol oxidase enzymes, whereas HHP favors the extraction of sugars and polyphenols and preserves TPC, thus enhancing the antioxidant activities and extending the product shelf-life [260].

Nevertheless, this technology is a promising and useful tool also for extracting polyphenols from grape skins and pulps and for limiting browning effects thanks to the inhibition of oxidative enzymes.

In red wine processing, due to the small cross-section of the homogenizer valve, it is not possible to directly treat whole grapes or pomace. HPH and UHPH can be applied to the unclarified must obtained after draining/pressing or after HHP extraction treatments. They perform their activity on the colloidal particles of the grape juice with a size of less than 500 µm, provoking their nano-fragmentation, increasing anthocyanin release [242].

The application of pressure below 200 MPa appears to be unsuitable for inactivating microorganisms since the inactivation rate at lower pressure seems to be more related to temperature increase [261]. Treatment of white and red grape must at just 200 MPa allows complete inactivation of indigenous yeasts, molds and lactic acid bacteria, even if other wild bacterial species may resist (30–500 CFU/mL); however, the implantation of selected yeast used for fermentation is favored, and the fermentation kinetics result faster [262]. Increasing the pressure up to 300 MPa, a complete inactivation of wild yeasts, bacteria [263,264,265,266] and even spores [267] may be achieved, with a significant reduction of sulfur dioxide needed in the pre-fermentative step. An interesting aspect of this technology is that the increase in temperature during the treatment, up to 98 °C inside the valve, is kept for a maximum of 0.2 s, allowing the preservation of grape must from thermal damage. HPH treatment of the must does not affect the basic enological parameters, in terms of pH and total acidity [262,264,266] whereas a slight increase in sugar content (up to 4 g/L) was observed [267]. It is interesting to note that the concomitant extraction of amino acids (up to 40 mg/L) and ammonia (up to 55 mg/L), compared to sulfited must [265], might also be useful to accelerate fermentation kinetics.

Especially in red winemaking, the application of HPH (or UHPH) has an important and significant impact also on the extraction of phenolic compounds. The increase in pressure (from 50 to 200 MPa) and especially the number of treatment cycles (1, 3 and 5 cycles at 200 MPa) cause a decrease in particle size and intense cell breakage and disruption, which determines not only microbial inactivation, but also the rupture of grape skin fragments. This favors better colloidal stability, possibly related to the decrease in particle size, raises the release of total polyphenols and anthocyanins and improves color stability over time [263,266,268], with color intensity and hydroxycinnamic acids content, in some cases, higher than in sulfited must [265].

The optimum conditions for obtaining the maximum extraction of phenolic compounds and flavonoids and the highest total antioxidant activity in red grape juice seem to be 550 MPa, 44 °C applied for 2 min, for pressure, temperature and processing time, respectively [269]. Similarly, a good extraction of anthocyanins from blueberry juice may also be reached with an inlet temperature of 4 °C and a pressure of 300 MPa, together with a higher stability during cold storage linked to the inactivation of PPO, up to 80% [270]. The higher color intensity and stability obtainable by HPH treatment depend on several factors: (i) the enhanced extraction of total polyphenols and higher concentration of acetylated anthocyanins (+9.3%) that contribute to the development of bluish-red hues highly valued by consumers [266]; (ii) the inactivation of polyphenol oxidase enzymes, even up to 90%, in pressed white grape must [265] consequentially preserving it from browning [263] and increasing the antioxidant activities of the must from 106% to 156% compared to the untreated sample [266,267]; (iii) the grapevine varieties [256] and finally (iv) the strains used for fermentation [264].

From the aroma and sensory point of view, HPH does not compromise the sensory profile of the resulting wines, compared to the untreated samples [262]. However, more recent knowledge advances highlight a better aroma quality and global perception, with more intense floral and fruity notes perceived in wines obtained by UHPH-treated must, linked to a lower concentration of higher alcohols and volatile acids, and higher content of varietal thiols [263,264] and esters [265,266].

### 4.4. Microwaves

#### 4.4.1. Mechanism of Action

Microwaves (MWs) consist of electromagnetic waves with wavelengths ranging from 1 m to 1 mm and frequencies between 300 MHz and 300 GHz, with industrial plants operating at frequencies ranging from 915 MHz to 2.45 GHz [271].

Microwave-assisted extraction (MAE) enhances the release of intracellular compounds from plant tissues through the rapid conversion of electromagnetic energy into heat. Unlike conventional heating, which relies on heat transfer from the surface inward, microwaves generate heat directly within the tissues via dielectric heating, resulting in rapid and volumetric warming of the plant matrix [272,273].

The heating process is mainly driven by two mechanisms: dipole rotation, in which polar molecules such as water continuously realign with the oscillating electromagnetic field, and ionic conduction, which involves the movement of dissolved ions within the cellular fluid. Both phenomena generate heat inside the cells, leading to a rapid increase in intracellular temperature [272].

As a consequence, intracellular water expands, and pressure builds up within cellular compartments, particularly the vacuoles. The resulting mechanical stress increases membrane permeability and may cause partial disruption of vacuolar membranes and cell wall structures. These changes reduce mass-transfer resistance and facilitate the release of intracellular compounds into the surrounding medium [273].

#### 4.4.2. Impact on Wine Quality

In winemaking, MWs represent another interesting emerging technology for accelerating the maceration step, thanks to the extraction of less astringent tannins from skins, the TPC and the enhanced formation of stable pigments without affecting wine quality and stability [274]. Compared to heat maceration, approximately 10% higher juice extraction yield has been achieved due to the more substantial intracellular damage at the skin cell level; furthermore, a greater content of yeast assimilable nitrogen is released in the treated must [275]. The higher amounts of phenolic compounds, like catechin and resveratrol, extracted by MWs treatment allow for an increase in the antioxidant power of musts and consequently of wines, compared to traditional techniques such as enzyme addition or thermovinification [276,277]. By treating Pinot noir grape must, characterized by lower anthocyanins and possibly tannin content compared to other red grape varieties [278], the amount of total phenolic compounds, anthocyanins and tannins in the must obtained by MW-assisted extraction was significantly higher than in the untreated control, even after 18 months of aging [279]. However, the improved wine color related to enhanced content of polymeric pigments depends also on viticultural practices [280] and grape ripeness [281], vintage, vinification techniques [282,283] and grape variety [284,285]. The application of MW for grape processing also allows for reducing the population of spoilage microorganisms and the amount of sulfur dioxide needed, consequently determining a shorter lag phase and faster fermentation kinetics [279], also linked to an enhanced extraction of yeast assimilable nitrogen [275]. A higher extraction of both free and bound volatile compounds in must treated with MW was also observed, together with an increase in the amount of higher alcohol and acetate esters that confer pleasant floral and fruity notes to the wine [286,287]. A further improvement of wine quality and stability might also be reached thanks to the ability of MW to enhance the activity of β-glucosidase up to 12.54% [288], while reducing the polyphenol oxidase activity by 39.58% compared to untreated enzymes in a model solution [289].

Despite the positive effects of MW treatments on enhancing polyphenol extraction, shortening the maceration step and speeding up the fermentation, with consequentially improving the overall wine quality and stability, this technology has not yet been authorized by OIV for processing grapes or must. Some industrial plants are already available for food processing, consisting of tunnels or ovens generally used for cooking, pasteurization, sterilization and defrosting, designed also for in-line production. Further investigations are needed to implement the current scientific knowledge for winemaking applications, as well as to standardize the processes and scale up this technology at the winery level.

### 4.5. Ohmic Heating (OH) and Moderate Electric Field (MEF)

#### 4.5.1. Mechanism of Action

Ohmic heating (OH) is based on the application of an electric field around 20–100 V/cm, with a frequency ranging from 50 Hz to 30 kHz, current intensity up to 5000 A/m^2^ and maximum power of 480 kW. When the electric field is applied, the electric current passes through the food matrix, determining fast heating (Joule heating). The thermal effect is the main mechanism of action of OH; however, based on the intensity of the electric field and energy input, the OH treatment may give a greater thermal effect or a higher cell permeabilization, known as electroporation. Among OH technologies developed during the last decades, moderate electric field or MEF (<1000 V/cm), ohmic-PEF (>1 kV/cm) and conventional PEF (1–40 kV/cm) may combine thermal effect and electroporation, in a different and more or less intense way [290].

#### 4.5.2. Impact on Wine Quality

A moderate electric field has been introduced as a non-thermal technology for food processing, also including extraction of bioactive compounds from grape by-products [291], representing an interesting and promising tool for enhancing the polyphenols extraction yield and potentially reducing the time necessary for maceration in red winemaking.

Even if the research on the use of ohmic heating for processing grape must is quite limited, several studies have been carried out to assess the effectiveness of this technology for improving the extractability of bioactive compounds from different food matrices and by-products.

At laboratory scale, the study on OH-treatment of red grape pomace, aimed at the recovery of bioactive compounds, revealed that if applied for a short time and in the high-intensity mode (30 V/cm, 25 kHz, 100 °C for 13 s), higher extraction yield of both total polyphenols content and anthocyanins was obtained, compared to conventional heating [292]. OH also allows higher energy savings, thanks to the shorter treatment time (up to sevenfold lower), while ensuring higher extraction yield, low thermal damage to the phenolic fraction and greater antioxidant and antimicrobial activities [292,293]. The extractability also depends on the treatment intensity [294,295], the presence of solvents and concentration, and grape variety [296].

When increasing the treatment intensity, the application of a moderate electric field (70 V/cm, 25 kHz, 100 °C for 30 s) led to a higher extraction yield of total polyphenol content, especially anthocyanins, up to 120% and 60% respectively, compared to conventional heating or low intensity OH treatment, thanks to an enhanced permeabilization of cells, without effective thermal damage [295]. A further increase in the extraction rate of polyphenols from red grape pomace was also observed when the treatment was performed in hydroalcoholic solution [297]. This evidence is particularly interesting for winemaking applications, since it might promote the extraction of polyphenols from OH-pretreated grape must, especially when maceration during fermentation is performed with the support of the solvent/extractive capacity of ethanol. However, even if not yet tested on grapes, a loss of bioactive compounds extracted from fresh parsley tended to occur when the intensity of OH treatment increased [294].

Ohmic heating may additionally enhance the extraction yield when combined with other emerging technologies. The maximum extraction of TPC from cornelian cherry was found in less than 60 min when OH was performed before sonication, with an increase in TPC of about 13% and 37% compared to conventional maceration and ultrasound alone [298]. In addition, the concentration of monomeric anthocyanins also increased (up to 50%) when OH was performed before microwave-assisted extraction [299]. This enhancement in extractability is mainly due to damage and cell disruption induced by OH, thus determining a faster release of intracellular components when US or MW were then applied, with a significant reduction in time and energy consumption, also in comparison with other emerging, non-thermal technologies [295].

Junqua et al. have evaluated the application of OH for pre-treating must under winemaking conditions [300]. No effect on fermentation kinetics was observed; on the other hand, the must and the resulting wines showed higher total polyphenol index and antioxidant power. However, the lower concentration of tannins and similar anthocyanin content compared to conventional thermal heating—both immediately after the treatment and during fermentation -could be of interest, since OH treatment might reduce the maceration time, whilst limiting the extraction of more astringent tannins from seeds that instead might occur during a more prolonged period of contact, typical of conventional maceration [5]. Higher concentrations of ethyl and acetate esters in wines obtained from OH-pretreated musts were also observed, with more intense fruity and floral notes, compared to control and wines obtained by conventional thermal treatment [300].

Another interesting aspect is related to the reduction in wild microorganisms [252], together with a significant inhibition of polyphenol oxidase and peroxidase that, under certain processing conditions, may be completely inactivated [294,301,302,303,304].

The available scientific evidence highlights the great potential of this technology in extracting several key compounds, with possible shortening of maceration time and lowering energy consumption compared to conventional heating or other emerging non-thermal technologies [292]. Nevertheless, like microwaves, ohmic heating is currently not applied at winery scale due to some limitations, first of all the limited studies to date carried out about its potential applications in winemaking. Furthermore, an improved understanding of key parameters playing a central role in the process effectiveness, particularly in the grape matrix, is needed at laboratory and pilot-scale, to allow optimization of the processing conditions. Since the results to date are still too variable and unpredictable, the potential of the treatments is thus not yet sufficiently understood.

## 5. Conclusions

By summarizing current knowledge on traditional and advanced maceration techniques in red winemaking, and on non-thermal emerging maceration technologies, this work offers a valuable comprehensive framework not only for practical benefits but importantly, also for aiding researchers to highlight areas where evidence remains insufficient and where future research efforts should be concentrated.

This review underscores the importance of addressing multiple interacting key factors and the complex nature of extraction mechanisms that affect the success and applicability of various maceration techniques. It also highlights the need for revisiting and conducting further research to improve the understanding, optimization, predictability, and longevity of their effects under different grape, processing, and overall winemaking conditions. Current drawbacks of existing research reports, such as substantial variation in experimental designs, uneven control (reference) bases for comparison, variations in applied analytical—chemical and sensorial methods, and different sampling points and times of observation, complicate the understanding of maceration processes beyond the unavoidable challenges related to cultivars, maturity levels, and geoclimatic origins specifics. In addition, while the body of chemical research is progressing, sensory evidence remains fragmented and comparatively scarce, often limiting the interpretation of the significance of observed compositional changes.

Future studies should therefore prioritize carefully designed and comparable experimental protocols, appropriate reference controls, advanced analytical methodologies, and validated sensory approaches with well-defined descriptors, together with longer-term evaluations extending beyond alcoholic fermentation into bottle aging. Particular attention should also be given to process scalability under industrial conditions and the identification of reliable markers for wine style and quality prediction. Such approaches would support more robust, evidence-based recommendations and facilitate the adoption of maceration strategies tailored to specific production objectives and emerging challenges.

## Figures and Tables

**Table 1:** Comparative overview of traditional and modified maceration strategies according to winemaking objectives, and generalized expected wine characteristics.

Winemaker’s Objective		Technique		Key Extraction Mechanism		* Generally Expected Key Wine Characteristics
Fresh, fruit-driven wines with the potential of early consumption		Pre-fermentative cold maceration		Selective aqueous diffusion under low temperature		Enhanced varietal expression, fresh fruit profile, softer tannins
		Carbonic maceration (direct pressing)		Intracellular fermentation under anaerobic conditions		Strawberry/cherry notes, lighter color and body, softer tannins
		Cryoextraction		Ice crystal-induced cell disruption and selective skin extraction		Greater color intensity, fuller fruit expression, softer texture
Balanced wines adaptable to diverse stylistic goals		Classical maceration with alcoholic fermentation		Progressive hydroalcoholic extraction driven by increasing ethanol concentration		Balanced color, structure and aroma profile
Age-worthy wines, great tannic structure, and long-term color stability		Post-fermentative extended maceration		Prolonged hydroalcoholic extraction promoting tannin diffusion and pigment polymerization		Enhanced color stability, tannin structure, sensory complexity, and aroma maturity, fuller body
		Whole-bunch fermentative maceration		Hydroalcoholic extraction combined with stem-derived compounds and partial intracellular metabolism		Enhanced texture, complexity, floral and spicy notes and perceived sweetness
Handling large volumes of entry-level wines with rapid winery turnover/ Vintage correction under high yields, incomplete maturation, rot incidence/sanitary challenges		Thermovinification		Thermal permeabilization		Darker fruit character, enhanced palate and reduced vegetal notes
		Flash release (détente)		Thermal treatment followed by sudden pressure release		Increased color density, rounder palate/stronger astringency
Extraction intensification under limited maceration time		Accentuated Cut Edges (ACE)		Increased skin surface area and mass transfer efficiency		Improved structure and color intensity

* Outcomes may vary depending on grape cultivar, fruit maturity, process conditions, fermentation management, and maceration duration.

**Table 2:** Comparative summary of key chemical and sensory impact of traditional and modified maceration techniques.

Technique	Anthocyanins Extraction	Color Intensity/ Stability	Flavan-3-ol and Tannin Profile	Astringency	Fresh Fruit	Ripe Fruit	Cooked Fruit/Jam
Pre-fermentative cold maceration	↑↔	↑ better color in musts, often carried into wine/↔	Skin-dominated, LMW	↓	↑↑	↔↓	–
Classical maceration with alcoholic fermentation	initially ↑	↑/↑	Skin and seeds-derived, increasingly polymerized	↔ ↑	↑	↑	–
Post-fermentative extended maceration	↔ ↓	↔ ↑/↑↑	Increasing seed contribution, polymerized	↑↑	↓	↑	– ↔
Whole-bunch maceration with alcoholic fermentation	↔	↔/↑	Skin-, seed- and stem-derived	↑↑	–	↔↑	–
Carbonic maceration	↔	↓/↓↔	Predominantly skin-derived, LMW	↓	↑↑	↓	–
Cryoextraction	↑	↑/↔	Predominantly skin-dominated	↓ ↔	↑	↓	–
Thermovinification	↑	↑/↔	Mixed, enhanced skin release with some seed contribution	↔ ↑	↓	↑	↔ ↑
Flash release	↑↑	↑/↔ ↑	Mixed; more skin-derived, polymerized; seeds-derived variable	↔ ↑	↔	↔ ↑	↔ ↑
ACE Accentuated Cut Edges	↑	↑/↔ ↑	Skin-derived, fragmentation-dependent	↔↑	↔	↔	↔ –

Arrows indicate generalized trends synthesized from the literature and should be interpreted as indicative rather than quantitative, as responses may vary with grape cultivar, maturity, vintage and winemaking conditions. Legend: ↑ generally associated with higher levels or greater expression; ↑↑ markedly higher levels or expression; ↓ generally associated with lower levels or expression; ↔ no consistent trend or variable response; – no or insufficient evidence; LMW; low-molecular-weight.

**Table 3:** Comparative technological fingerprint of modified traditional maceration techniques.

Attribute		Cryoextraction		Thermovinification		Flash Release		Accentuated Cut Edges (ACE)
Driving force		Cryogenic		Thermal		Thermo-mechanical		Mechanical
Winery throughput		Low		High		High		Moderate
Energy intensity		High		High		Very high		Low
Technology maturity		Established- intermediate		Established		Intermediate-emerging		Intermediate-emerging
Available research evidence in red winemaking		Low		Low-Moderate		Low		Low
Potential relevance for climate-adaptive winemaking—possible future research orientation		Enhanced preservation of freshness-related aroma profiles under climate warming conditions		Compressed harvest windows and winery processing constraints associated with accelerated grape maturation		Multifaceted adaptation to maturity, sanitary and logistical challenges		Mitigating loss of phenolic maturity and extraction efficiency caused by earlier harvest dates adopted to preserve acidity

**Table 4:** Main advantages, impact on wine quality, and OIV status of the different emerging technologies.

Emerging Technology		Advantages		Impact on Wine		OIV Status
Ultrasound		Accelerated extraction due to cavitation; reduction in maceration time from days to minutes; low thermal damage; short treatment time up to 120 min; reduction in spoilage microorganisms		Increase in total phenolics, anthocyanins and polymeric pigments after fermentation and aging; higher aging potential of wine; higher color intensity and stability; no significant impact on wine aroma profile		Approved for enhancing extraction during maceration
Pulsed electric fields		Accelerated extraction due to electroporation; reduction in maceration time; very short treatment time (up to a few seconds)		Increase in tannins and anthocyanins concentration; higher color intensity and stability over time; extraction of varietal aroma precursors		Approved for enhancing extraction during maceration
High hydrostatic pressure		Complete inactivation of spoilage microorganisms and enhanced extraction due to mechanical damage to the cells; low thermal damage; short treatment time from 2 to 25 min; reduction in oxidative enzyme activity; better implantation of fermentation starters		Increase in tannins and anthocyanins concentration; higher color intensity; no or slight impact on basic enological parameters; slight increase in higher alcohols and esters, better sensory perception of wine		Approved for inactivation of spoilage microorganisms
High pressure homogenization		Complete inactivation of spoilage microorganisms and enhanced extraction due to mechanical damage to the cells; low thermal damage; short treatment time; reduction in oxidative enzymes activity; better implantation of fermentation starters		Reduction in colloidal particle size and protein instability; higher antioxidant activity of must; no impact on basic enological parameters; higher concentration of total polyphenols and acetylated anthocyanins; slight increase in esters concentration and varietal thiols; better aroma quality and global sensory perception		Approved for inactivation of spoilage microorganisms
Microwaves		Accelerated extraction due to intracellular heating; reduction in maceration time up to 72 h; short treatment time; reduction in oxidative enzymes activity; better implantation of fermentation kinetics		No impact on basic enological parameters; higher total polyphenols index, total tannins and anthocyanins, formation of more stable pigments, higher color intensity; greater concentration of higher alcohols and acetate esters in wines; improved sensory profile		Not approved
Ohmic heating		Accelerated extraction due to both heating and electroporation (in relation to treatment intensity); higher cell permeabilization; reduction in maceration time; low processing time and energy consumption; significant reduction in spoilage microorganisms and oxidative enzyme activity		No effect on fermentation kinetics Higher total polyphenol index and antioxidant capacity Higher concentration of esters Better sensory profile, less vegetal and fruitier notes		Not approved

## Data Availability

No new data were created or analyzed in this study. Data sharing is not applicable to this article.

## Supplementary Materials

The following supporting information can be downloaded at: https://www.mdpi.com/article/10.3390/foods15142571/s1, Table S1. Comparative overview of conventional wine maceration strategies: traditional approaches and common modifications. Table S2. Main results of the application of emerging technologies in pre-fermentative maceration.

## Abbreviations

The following abbreviations are used in this manuscript:

ACE	Accentuated cut edges
AF	Alcoholic fermentation
CE	Cryoextraction
CFU	Colony-forming unit
CM	Carbonic maceration
CM+F	Classical maceration with fermentation
FR	Flash release
HHP	High hydrostatic pressure
HMW	High-molecular-weight
HPH	High-pressure homogenization
HPP	High-pressure processing
LMW	Low-molecular-weight
MEF	Moderate electric field
MWs	Microwaves
OH	Ohmic heating
OIV	International Organization of Vine and Wine
PA	Proanthocyanidins
PEF	Pulsed electric field
POD	Peroxidase
PostFEM	Post-fermentative extended maceration
PPO	Polyphenol oxidase
PreFCM	Pre-fermentative cold maceration
TPC	Total phenolic content
TV	Thermovinification
UHPH	Ultra-high-pressure homogenization
US	Ultrasound
WBM+F	Whole-bunch maceration with fermentation
