# Supplementary material for: Traditional and Emerging Maceration Techniques in Red Winemaking: Extraction Mechanisms Shaping Phenolic Composition, Volatile Profile, and Sensory Expression
Source: Foods. 2026 Jul 22;15(14):2571. doi: 10.3390/foods15142571 (PMC13409729; doi:10.3390/foods15142571)
Supplement: Supplementary file 1 [file foods-15-02571-s001.zip › foods-4396382-supplementary.pdf]

Supplementary Material Table S1. Comparative overview of conventional wine maceration strategies: traditional approaches and common modifications.

Attribute	Pre-fermentation cold maceration (PreFCM)	Classical maceration with fermentation (CM+F)	Post-fermentation extended maceration (PostFEM)	Whole bunch maceration with fermentation (WBM+F)	Carbonic maceration (CM)	Cryoextraction (CE)	Thermovinification (TV)	Flash détente / Flesh release (FR)	Accentuated Cut Edges (ACE)
Principle	Low-T maceration of intact or crushed grapes in the absence of active fermentation; strictly pre-alcoholic, aqueous extraction phase, favoring water-soluble, skin-localized compounds.	Standard AF conducted with grape solids present throughout; solvent polarity rises continuously as EtOH increases → compound-specific extraction.	Extension of skin and seed contact with must after completion of AF aiming to further extract and/or transform phenolics and enhance tannin structure.	AF in the presence of partially or fully intact grape bunches including stems, integrated with partial intracellular/carbonic metabolism within intact berries and extraction of stem-derived compounds.	Anaerobic maceration of intact grape clusters under a CO_2-rich atmosphere; intracellular fermentation occurs within berries prior to pressing and completion of AF.	Freezing whole grapes or pomace to sub-zero T, followed by partial thawing before pressing or short pre-AF maceration to enhance selective extraction.	Rapid pre-AF heating of crushed grapes/must to accelerate phenolic extraction, inactivate PPO and spoilage microbes, followed by pressing, cooling and AF in liquid phase or in presence of solids.	Instantaneous cell disruption by rapid pre-AF heating of crushed grapes followed immediately by cooling under strong vacuum, causing expansion/ “explosion” of berry tissues → extraction.	Mechanical fragmentation of grape skins, involving cutting/shredding to ↑ total surface area and number of open or accessible skin edges with the aim to enhance the extraction of skin-derived phenolics and aroma precursors.
Typical conditions	T ≈ 4–15 °C; 1–7 d; lightly crushed grapes, aqueous medium; protective SO_2 addition; delayed AF; low T slows enzymatic oxidation; pectolytic enzymes (optional).	T ≈ 20–30 °C; 5–21 d total maceration; yeast inoculation (commercial starters or spontaneous); EtOH ↑ 0 ≈12–14% v/v; CO2 protection against oxidation; cap management 1–3×/d.	T 15–30 °C; 7–30+ d post-AF; (EtOH) ≈12–14% v/v; passive or gentle pump-over; loss of protection associated with active AF (CO2), O_2 management critical.	≈10–100% whole bunch inclusion = intact clusters (stems retain) fermented alone or mixed with destemmed/crushed grapes; otherwise, standard red wine AF conditions.	Undamaged, healthy grape clusters (with stems) placed in sealed CO_2-filled vessels; T/duration approaches vary: e.g. ≈5–8 d at 30–32 °C vs ≈15–20 d at 15 °C; competition of AF after pressing with spontaneous or commercial yeast addition.	T ≈ −1 to −8 °C+; freezing achieved using mechanical refrigeration and/or cryogenic agents (e.g. liquid CO_2, liquid N_2, dry ice), followed by partial thawing of grapes before pressing/AF.	Crushed grapes/must heated to ≈50–80 °C (≤ 85 °C) for 1-10 min+ in heat exchangers or similar, while maintaining solid/liquid phase contact, then pressed, rapidly cooled (≈35–40 °C), often clarified before AF (with/without solids).	Destemmed/crushed grapes heated to ≈80–95 °C for ≈. 5-15 min, then transferred to vacuum/ expansion chamber (≈20–50 hPa), sudden pressure drop cools the mass to ≈30–35 °C; pressing before or after short maceration, with AF in liquid phase or with solids present.	Applied after crushing using high-shear/blade-type devices on pomace; grape skins are cut/shredded into small fragments (≈10 % of their original size), ideally without damaging the seeds; generally short maceration followed by AF.
Primary driver of extraction & some key co-factors	Aqueous diffusion across osmotically disrupted cell walls; low EtOH limits seed tannin extraction; ↑ extraction of aroma precursors.	Progressive ↑ EtOH (polarity shift) + ↑ T + CO_2 agitation; yeast enzymatic activity; mechanical cap management.	Hydroalcoholic medium and ↑ contact time → ongoing passive diffusion and desorption of phenolics, especially seed-derived tannins; pigment–tannin	Conventional hydroalcoholic extraction during AF, however, upgraded with extraction of stem-derived compounds and sometimes partial intracellular fermentation.	Anaerobic conditions trigger metabolic shift within intact berries from respiration to intracellular fermentative metabolism; limited AF in free-run juice at the	Ice-crystal formation rupturing pectocellulosic cell walls, increasing porosity and release of skin phenolics and aroma precursors, with low T	Thermal disruption → ↑ cell permeability → rapid release of intracellular phenolics (particularly anthocyanins); PPO inactivation; extraction patterns further shaped by	Combined thermal disruption of cell walls/ + rapid pressure-induced expansion of intracellular water → rapid expansion and disintegration of berry tissues; ↑ in effective skin surface area in	↑ cut surfaces and exposed skin edges facilitate mass transfer and diffusion of compounds from disrupted skin tissues into the must/wine, thereby accelerating

			condensation and other polymerization/precipitation reactions proceed. Flavor compounds released from yeast autolysis.		bottom, anaerobic phase followed by conventional AF.	suppressing AF and inhibit oxidation.	T/time of subsequent skin/no skin contact.	contact with juice; PPO inactivation.	extraction kinetics and often enhancing extraction efficiency.
**Phenolic impact (anthocyanins, tannins, TP; other phenolic groups and key grape/process factors discussed in the text).**	Often ↑ monomeric anthocyanins and skin-derived flavan-3-ols; limited seed-derived tannin extraction due to low T and low/no EtOH conditions; ↑ color-related parameters in musts; sometimes ↑ TP. However, outcomes are often ↔.	Broad phenolic extraction: early ↑ anthocyanins and skin PAs; later ↑ seed PAs as EtOH rises; more polymeric pigments toward end of fermentation.	Normally gradual ↓ in free monomeric anthocyanins; ↑ tannins (particularly seed-derived) and TP; polymeric pigments and HMW tannin fractions tend to ↑ to a point, but excessive duration may favor precipitation and loss.	↑ stem-derived flavan-3-ols, tannins, copigmentation and color stability; anthocyanins and TPC ↔; may ↑ astilbin; shifts tannin composition away from seed-derived tannins; stem maturity- and inclusion rate-dependent effects.	Often ↓ anthocyanins, TP and color related characteristics, but in some studies ↔; single study reported ↑ vitisins A and B, linked with improved long term color stability.	Tends to ↑ anthocyanins and, in some cases, combined tannin–anthocyanin pigments, reported effects on TP inconsistent (↔), indicating more selective, skin-focused extraction in some protocols.	Generally, ↑ anthocyanins and skin tannins; anthocyanin degradation may occur if T > ≈ 70 °C; ↓ galloylated seed tannins; however, these effects and TP results sometimes ↔.	Generally, ↑ main phenolics and color density, polymeric pigments may ↑, but anthocyanin and TP gains ↔ or not maintained after maturation; responses may vary also with whether musts are pressed immediately or kept on skins after FD.	May ↑ color intensity, non-bleachable pigments, skin-derived monomeric phenolics and tannins; prolonged ACE may ↑ anthocyanin extraction.
Aroma impact	↑extraction/ preservation of varietal precursors (glycosidic terpenes, thiol, norisoprenoid precursors); ↓ volatilization losses at low T; ester profile may be shaped by different organic and mineral nitrogen ratio. Off-note risk low when SO_2 or appropriate bioprotection and hygiene are adequate.	Aroma development dominated by yeast metabolism (esters, higher alcohols, sulfur compounds); moderate varietal precursor release; T management (< 25 °C) critical for aroma retention; appropriate AF management crucial to prevent off-note risk.	↓ ester- and terpene related freshness over time; tends to ↑ β-damascenone and other evolved norisoprenoid notes; prolonged contact favors more savory/matured characters; occasional reductive or oxidized nuances if O_2 and hygiene are poorly controlled; overall aroma effects tend to be subtle, context-dependent.	May ↑ aroma complexity and diversity also via partial intracellular fermentation; higher stem inclusion may ↑ methoxypyrazines, some C6 compounds, β-damascenone, eugenol, guayacol and ethyl cinnamate.	Generally, associated with ↑ esters and acetates, may ↑ ethyl cinnamate, ethyl dihydrocinnamate and 3-mercaptohexanol. Indirect effect: may contribute to ↓ malic acid and alcohol concentration (changing matrix effects and microbial metabolism).	↑ varietal aroma compounds and glycosidic precursors; may cause selective shifts in fermentative ester and higher-alcohol profiles in final wines compared to CF+M; very limited data suggest possible ↑ in some volatile phenols, but remaining below fault-perception levels.	May ↑ some fermentative esters under specific cultivar/processing conditions; however, higher T generally ↓ varietal terpenols/ norisoprenoids and may ↑ the risk of formation of heat-related unwanted aroma.	Tends to ↓ green/vegetal notes (e.g. methoxypyrazines), but can also ↓ some varietal terpenes and norisoprenoids; fermentative esters, higher-alcohol profiles may shift in cultivar-dependent ways. Possibility of heat-related aroma.	Selective; limited evidence; ACE may ↑ some ethyl esters, higher alcohols, fatty acids and terpenes; acetate esters and certain branched-chain ethyl esters may ↓.

Sensory outcome (typical tendencies)	In general, deep initial color; ↑ fresh, fruity aroma profile; soft, smooth tannin texture and often ↓ astringency compared to other conventional approaches; however, the final wine characteristics remain strongly influenced by subsequent AF and other enological decisions.	Sensory outcomes hard to generalize (broad stylistic range, yeast metabolism), but wines usually show balanced color, structure and aroma; differences in color and mouthfeel mainly reflect maceration time, T and cap management rather than CM+F itself.	↑ deeper, more persistent color; ↑ body and structural complexity; generally, ↑ astringency, tannin grip and sometimes bitterness at ↓T; warm PostFEM may ↓ astringency, ↑ body and sweetness; ↓ fresh-fruit and floral notes. Aroma continues to evolve, more developed, complex notes over time.	Associated with ↑ tannin structure, mouthfeel complexity and deeper color; may ↑ sweetness and floral, bitter almond, spicy/clove, vegetal, and green notes.	Typically, → fruity and more intense aroma profile; particularly of soft red fruits, e.g. ↑ strawberry, cherry, and confectionary-like notes. Generally lighter body, softer tannins and ↓ astringency, but longer CM/AF may ↑ complexity and structure.	Limited sensory evidence; sometimes → fuller, more intensely colored wines with ↑ soft-fruit/ cherry/ liquorice/ cinnamon notes and ↓ astringency, ↑ dryness in some cultivars; overall quality not consistently ↑ and coldest treatments may ↓ balance.	Wines tend to be more intensely colored, fruitier with ↑ dark red fruit (blueberry, blackberry); and may have ↓ vegetal, spicy, earthy/moldy character and ↑astringency. Overall sensory quality observations are not consistent.	Tends to give wines with red fresh-fruit or confectionery notes (if grape solids are present ↑ darker-fruit character); may ↓ green, floral, and savory notes; may express fuller body, greater astringency and sometimes bitterness.	May ↓ red fruit notes and ↑ dark fruit, confectionary-like, vanilla and earthy/dusty characters; color perception may be unaffected; bitterness/astringency may increase, but ↔ results on consumer tests.
Capital intensity / energy	Low–moderate; standard insulated tank with cooling jacket or refrigerated room, no other dedicated specialist equipment needed.	Low; standard open or closed fermentation tanks; universal winery infrastructure; reference baseline cost.	Low additional direct cost; but ↑ energy cost for warm PostFEM; same tanks as CM+F, requiring mainly extended tank occupancy and timing management; but prolonged tank use may reduce winery throughput.	Low; no specialized equipment required; eliminates destemming and crushing operations; minimal additional labor associated with handling whole clusters.	Moderate; requires sealed, pressure-stable vessels and CO_2 management; slightly higher technological demands than conventional fermentation.	High: requires cryogenic gases and/or freezing equipment, temperature-controlled storage, additional handling; ↑ gas and energy use vs standard PreFCM.	Moderate to high: requires heating and cooling systems and process control; energy/cost ↑ vs CF+M, but may be compensated partly by faster processing and ↑ throughput in some settings.	High: requires integrated heating, vacuum chamber, condensers, control systems; ↑ capital cost and energy use, although rapid extraction and shorter overall maceration can improve tank turnover.	Low to moderate: requires an additional mechanical fragmentation step (ACE device, high-shear mixer or similar) but no major thermal or cryogenic equipment; energy use modest.
Generally suited for	Warm-climate grapes at risk of over-extraction; fresh-fruit style reds; premium earlier-drinking wines, unique-styled premium wines.	All quality red wine production; adjustable across full stylistic range from light to full-bodied.	Structured, age-worthy styles where increased tannin integration is desirable; less suited to early-drinking, fruit-driven wines.	Premium red wines where greater complexity, texture, tannin structure and ageing potential are desired.	Fruit-forward, early-drinking wines when followed by direct pressing; longer CM with subsequent conventional red vinification → structured, complex wines with ageing potential.	High-quality reds, typically in limited lots, where extra color or distinctive sensory attributes are targeted (but not necessarily achieved), in wineries with reliable access to cryogenic infrastructure.	Primarily high-volume/mid-range wines requiring rapid anthocyanin/phenolics extraction, fast tank turnover and ↑ MO control, selectively applied in higher quality wines for targeted color or stylistic objectives.	Generally suited for under-ripe grapes; MO affected lots (PPO inactivation); high-volume reds needing rapid/high phenolic extraction and color density; style differentiation toward fruity, rounder, early-drinking reds.	Grapes needing enhanced phenolic and aroma extraction (e.g. low-tannin or cool-climate reds); structured reds without extensive maceration; flexible extraction tool in climate-adaptation winemaking.

Potential advantages	Selective extraction of skin-derived compounds under pre-fermentative conditions; ↓ risk of overextraction; flexible and easy-to-implement technique; compatible with a wide range of winemaking styles and grape varieties.	High flexibility across all wine styles; broad phenolic and aroma spectrum; base technology with no additional investments.	↑ color stability via polymeric pigments/co-pigmentation; ↑ structural complexity and longevity = improves ageing potential; warm PostFEM may add sweetness; no additional equipment required.	Easy to implement with minimal process modifications; offers stylistic flexibility and suitability across a wide range of cultivars.	Unique stylistic differentiation when fruity aroma profile is desired; flexible protocols for modulation ↓↑ of tannin harshness and astringency; reasonable investment and processing costs.	Efficient berry-level extraction; may have potential to ↑ color and structure without strongly ↑ harsh seed tannin; low-T phase limits oxidation, MO spoilage and premature AF; additional stylistic tool.	Rapid/efficient early extraction of skin phenolics; inactivation of PPO and spoilage MO; potential to stabilize color and ↑ process hygiene; ↑ harvest-time capacity by shortening time on skins, offer marketable wines from poor quality grapes.	Maximum extraction efficiency in minimum time; PPO fully inactivated; ↑ color and TP even from low-maturity fruit; vacuum step may simultaneously remove off-volatiles; high throughput; lag phase prior AF may be slightly shorter.	Rapid/efficient extraction of skin phenolics; potential ↑ color intensity/stability and wine structure; may enhance fruity/floral aroma expression; limited seed damage/ ↓ excessive harsh tannin extraction risk.
Potential limitations	Phenolic profile shifted toward skin-derived fractions; potential for lower seed-derived structure particularly vs. post AF prolonged treatments; elevated microbial risk if SO_2 and hygiene are inadequate; increased refrigeration time/load in the pre-fermenting stage.	Outcomes highly dependent on precise AF management (T, duration, cap handling, yeast/nutrition) and grape matrix; risk of over-extraction (notably of seed tannins) if maceration or cap management is excessively intensive or poorly controlled.	↑↓T determines ↑↓ seed tannin harshness, excessive astringency and bitterness if lasts too long (risk of overextraction); ↓ fresh-fruity character; risk of reductive/oxidative or other spoilage notes if oxygen exposure and hygiene are not well controlled; longer winery cycle and tank occupation.	Risk of excessive vegetal, woody or stemmy character; may ↑ compounds related to green notes; anthocyanin adsorption by stems may ↓ color intensity; reduced tank filling efficiency.	Requires healthy, undamaged grapes; mechanical harvesting may be unsuitable; risk of VA and spoilage if hygiene or CO_2 management are inadequate; often limits color density, structure and ageing potential.	Increased process complexity (equipment) and processing time; high energy demand/costs; potential ↓ fermentation kinetics due to must clarification or yeast stress; hygiene and microbial control remain critical during thawing/maceration; usually limited production quantity.	Requires specialized heating/cooling equipment, ↑ energy demand and processing costs; potential loss/modification of varietal aromas and fresh-fruity character due to heat exposure; risk of ↑ cooked/jammy or oxidative notes at excessive T; Heat can → precipitation and ↓ of some phenolics and aroma precursors.	Requires specialized equipment and higher processing costs; aggressive regimes (e.g. T > 85 °C) can promote cooked-fruit notes, strip or mask varietal aromas, and cause over-extraction with altered astringency/mouth feel. Limited data on sensory and long-term ageing performance.	Requires specialized equipment; overapplication risks excessive seed breakage → ↑ astringency /harsh tannins; accelerated extraction may ↓ extraction selectivity; possible ↓ fresh-fruity character; ↑ suspended solids may complicate cap management, clarification and AF dynamics; very limited sensory and long-term validation available.
Selected references (others and more details in the main text).	[18, 20, 22,25,35,36,46,54, 64]	[16,17,19,44,71,72,74,80,90,102,106]	[71,82,93,102,106, 137,139,142–144]	[39,110–112,114–117,119]	113,127,128,131,132,134]	[17,25,49,57,159,161,164,166,167]	[17,28,79,125,174–176,179,180]	[17,174,175,183–185,187–190]	[125,168–173]

Legend: ↑ increase; ↓ decrease; → no consistent change/variable results; → promotes; ≈, approximately. Abbreviations: T, temperature; EtOH, ethanol; d, day; TP, total phenolic content; HMW, high-molecular-weight; PA, proanthocyanins; VA, volatile acidity; PPO, polyphenol oxidase (used here to collectively denote oxidative enzymes); AF, alcoholic fermentation; MO, microorganisms

Supplementary Material Table S2. Main results of the application of emerging technologies in pre-fermentative maceration

Ultrasounds			
Variety	Operating Conditions	Results	Ref.
Monastrell	Pilot-scale system (MiniPerseo; Agrovin S.A., Alcazar de San Juan, Spain)<br>Power 2500 W, frequency 28 kHz, power density 8 W/cm2, maceration time 2 and 5 days<br>Power of 9000 W, frequency of 30 kHz, power density of 58.5 W/cm2, maceration time 3 and 7 days; US alone and in combination with pectolytic enzyme	Increase in grape polysaccharides content	[198–200]
Monastrell	Pilot scale system MiniPerseo (Agrovin S.A., Alcazar de San Juan, Spain)<br>Power 2500 W, frequency 28 kHz, power density 8 W/cm2, maceration time 3, 6, 8 days	Increase of total phenols, anthocyanins and polymeric anthocyanins, color intensity.<br>No impact on aroma compounds.<br>Better phenolic profile after fermentation and aging	[195]
Monastrell	Pilot-scale system MiniPerseo (Agrovin SA, Alcázar de San Juan, Spain)<br>Power 2500 W, frequency 20 kHz and 28 kHz, power density 8 W/cm2, maceration time 2 and 3 days	Higher color intensity, total phenol index, total anthocyanins and polymeric anthocyanins, at the end of maceration, fermentation and bottling, higher methanol content in bottled wines<br>No effect on pH, total acidity, ethanol, slight increase in K and Ca.<br>Higher aging potential of wine	[201,204]
Monastrell	Pilot-scale system MiniPerseo (Agrovin SA, Alcázar de San Juan, Spain)<br>Power 2500 W, frequency 20 kHz and 28 kHz, power density 8 W/cm2, maceration time 2 and 3 days	Increase of free and bound terpenes, free C6 alcohols and free benzenic compounds in wines<br>Increase of acids and esters, positive effect on higher alcohols and benzenic compounds, no significant changes in the concentration of sulfur compounds, pyrans and furans	[203]
Monastrell	Ultrasonic bath (Branson 8800 Ultrasonic cleaner, 25 L)<br>Power 280 W, frequency 40 kHz, alone and in combination with pectolytic enzyme (1 mL/hL), 90 minutes, T°C 18°C, maceration time 7 days	Increase of color intensity, total anthocyanins, total polyphenol index, polymeric anthocyanins; epigallocatechin; epicatechin gallate, especially in combination with enzyme. Better phenolic profile after fermentation and aging	[197]
Pinot Noir	Pilot scale, device ULTRON Unitronics (Dywity, Poland)<br>Power 380 W, frequency 40 kHz, T°C 23°C-25°C, maceration time 120 min	Reduction of total yeasts, bacteria and molds (90%)<br>Significant reduction of maceration time (from 6 days to 100 min)<br>Increase of color parameters (L*, a*, b*), total polyphenol content<br>Higher sugars, total acidity, YAN, FAN and NH4+, lower pH	[195]
Primitivo<br>Nero di Troia<br>Aglianico	Ultrasonic system Sonic Digital LC 1500 SD 25-P ultrasonic generator and Sonopush HD Double Twin 1500 Titanium transducer<br>Power 1500W, frequency 25 kHz, effective power 60 W/L	Increase of volatile compounds, also depending on cultivar	[202]
Pulsed electric fields (PEF)			
Variety	Operating Conditions	Results	Ref.
Garnacha,<br>Mazuelo,<br>Graciano	Discontinuous batch parallel chamber<br>Electric field strength: 2-10 kV/cm<br>Pulse duration and frequency: 50 pulses, 1 Hz<br>Pulse wave form: exponential decay<br>Total specific energy: 0.4-6.7 kJ/kg	Improved extraction of color, total phenolics and anthocyanins during maceration<br>Slightly lower tint (Abs 420/Abs 520) and more distinct red tones	[222]
Tempranillo	Discontinuous batch parallel chamber<br>Electric field strength: 5-10 kV/cm<br>Pulse duration and frequency: 50 pulses, 1 Hz<br>Pulse wave form: exponential decay<br>Total specific energy: 1.8-6.7 kJ/kg	Improved extraction of color, total phenolics, tannins and anthocyanins during maceration	[222]
Cabernet Sauvignon	Collinear chamber<br>Flow rate: 118 kg/h<br>Electric field strength: 5 kV/cm<br>Pulse duration and frequency: 3 µs, 122 Hz<br>Pulse wave form: square bipolar waves<br>Total specific energy: 3.67 kJ/kg	Accelerated maceration<br>Greater color intensity, anthocyanins content and total polyphenols in PEF treated wines. Differences maintained after 4 months<br>Higher mean sensory score for taste and astringency in PEF wine	[229]

Aglianico, Piedirosso, Nebbiolo, Casavecchia	Discontinuous laboratory batch system<br>Electric field strength: 1.5-3.0 kV/cm<br>Pulse duration and frequency: not reported<br>Pulse wave form: monopolar square waves<br>Total specific energy: 10-20 kJ/kg	Significantly greater release of polyphenols in Aglianico: +100% total phenolics, +30% anthocyanins, +20% color intensity<br>Results less relevant for Piedirosso, Nebbiolo and Casavecchia	[303]
Graciano, Tempranillo, Grenache	Collinear chamber<br>Flow rate: not reported<br>Electric field strength: 7.4 kV/cm<br>Pulse duration and frequency: 10-20 µs, 300-400 Hz<br>Pulse wave form: square bipolar waves<br>Total specific energy: not reported	Increased potassium content, higher color intensity, lower tonality and greater total polyphenolic index<br>Increased stilbene content (+200% in Tempranillo, +60% in Grenache and +50% in Graciano)<br>Effects more evident for cis- and trans-piceid than for resveratrol	[223]
Graciano, Tempranillo, Grenache	Collinear chamber<br>Flow rate: 400 kg/h<br>Electric field strength: 7.4 kV/cm<br>Pulse duration and frequency: 10-20 µs, 300-400 Hz<br>Pulse wave form: square bipolar waves<br>Total specific energy: not reported	Slight increase of potassium content<br>Enhanced aroma composition in Grenache: increased concentration of monoterpenoids, β-ionone, total esters and benzenoid compounds<br>No relevant effects on Tempranillo and Graciano<br>Few differences found between treatments for C6 compounds	[227]
Graciano, Tempranillo, Grenache	Collinear chamber<br>Flow rate: not reported<br>Electric field strength: 7.4 kV/cm<br>Pulse duration and frequency: 20 µs, 400 Hz<br>Pulse wave form: square bipolar waves<br>Total specific energy: not reported	Total phenolics and anthocyanins increased after PEF treatment during two vintages<br>Results more evident in Tempranillo<br>Higher concentrations in tartaric acid and potassium in PEF treated musts<br>Lower tonality and higher color intensity after PEF processing<br>Great reduction of maceration time	[224]
Garganega	Collinear chamber<br>Flow rate: 200 L/h<br>Electric field strength: 1.5 kV/cm<br>Pulse duration and frequency: 0-16 µs, 600 Hz<br>Pulse wave form: square-wave<br>Total specific energy: 11-22 kJ/kg	Increased pressing yield<br>Slight increase in wine dry extract<br>Reduction of volatile phenols in wine<br>Increase of varietal aroma precursors in grape juice	[226]
Rondinella	Collinear chamber<br>Flow rate: 250 L/h<br>Electric field strength: 1.5 kV/cm<br>Pulse duration and frequency: 0-10 µs, 400 Hz<br>Pulse wave form: square-wave<br>Total specific energy: 2-20 kJ/kg	Accelerated color and polyphenols extraction with respect to pectolytic enzymes<br>Increased wine color and phenolics, stable over 12 months<br>Increased anthocyanins, tannins and vitisin A content<br>Better evolution of the phenolic fraction during storage<br>PEF treated wines averagely perceived as more colored, full-bodied and structured during sensory evaluation<br>No detectable release of chromium, iron or nickel	[228]
Grenache	Collinear chamber<br>Flow rate: 2500±200 kg/h<br>Electric field strength: 4 kV/cm<br>Pulse duration and frequency: 3.7 pulses of 100 µs, in 90 ms<br>Pulse wave form: square-wave<br>Total specific energy: 6.2 kJ/kg	Higher content of tannins, anthocyanins and color in PEF processed samples at bottling<br>The effects of PEF remained evident after 24 months, during bottle or barrel ageing<br>PEF treatment allows to reduce maceration time from 6 to 3 days<br>Wines obtained by PEF were preferred during sensory test	[303]
Graciano, Tempranillo, Grenache	Collinear chamber<br>Flow rate: not reported<br>Electric field strength: 7.4 kV/cm<br>Pulse duration and frequency: 20 µs, 400 Hz<br>Pulse wave form: square bipolar waves<br>Total specific energy: not reported	Increased concentration of potassium and tartaric acid in PEF processed musts<br>Higher concentration of color, anthocyanins and total phenolics in PEF processed musts; greater color and lower tonality<br>Comparable or slightly higher color intensity in PEF treated wines with 2 days of maceration and untreated wines macerated for 5 days<br>Higher polymerization index in PEF wines	[225]
High hydrostatic pressure (HHP)			
Variety	Operating Conditions	Results	Ref.
Blueberry pomace	300-500 MPa for 3-15 min at 20°C	Extraction of anthocyanins	[246–248]
Grape pomace	300-500 MPa for 3-10 minutes at 22-30°C<br>50, 100 and 200 Mpa, 30 min<br>600 MPa, 70 °C for 1 h	Extraction of anthocyanins and tannins	[249–251]
Grape juice	500 MPa for 5 min, 25°C	Significant reduction of yeasts, bacteria and molds<br>Reduction of PPO (30%)<br>Slight increase in anthocyanins; no significant change in color and chemical profile	[252]

Grapes and various fruits	600-900 MPa	Reduction of PPO and POD	[258,259]
Pinot noir	551 Mpa for 10 min	Complete inactivation of yeasts and bacteria<br>Better implantation of fermentation starters	[244]
Tempranillo	200, 400 and 550 MPa for 10 min, T°C 20°C	Complete inactivation of yeasts; reduction of bacteria<br>Increase in total anthocyanins, color intensity and total polyphenol index; higher concentration of methanol and ethanol, small increase in acetoin and 2,3-butandediol, increase of higher alcohols and esters at lower pressure<br>Better sensory perception	[243]
Tempranillo	400 MPa for 10 min, T°C 20°C	Complete inactivation of yeasts<br>Better implantation of fermentation starters	[245]
Mouchtaro	200-600 Mpa, 5-15 min, 20°C	No effect on basic enological parameters<br>Reduction of total polyphenols, proanthocyanidins, anthocyanins and antioxidant activity at highest pressure and longer time<br>Increase in mean degree of tannin polymerisation and galloylation<br>Reduced volatile acidity<br>Slight impact of volatile compounds<br>More balance, higher overall quality, more aging sensory characteristics	[254]
Beibinghong	300 MPa for 2 min	Increased copigmentation	[255]
Cabernet Sauvignon<br>Merlot	407 MPa, 25 min, T°C 26°C	No or slight effect on basic enological parameters (pH, TA, sugars)<br>Increase in color, total phenols, anthocyanins, flavonols, flavanols, and resveratrol<br>Reduction of PPO	[256]
Trebbiano	600 and 900 MPa for 10 min	Reduction of PPO at higher pressure and longer time	[257]
High pressure Homogenization (HPH/UHPH)			
Variety	Operating Conditions	Results	Ref.
Red sugarcane	High Pressure Processing machine (Model QFP 2L-700 Laboratory Food Processing System, Avure Technologies, USA<br>300 MPa, 2 and 5 min, 25°C	No significant changes in color parameters<br>Inactivation of microorganisms<br>Reduction of PPO activity at longer processing time<br>Reduction in total polyphenols content and antioxidant activity	[260]
Parellada (white)<br>Trepat (red)	high-pressure homogenizer (model FPG 11300, Stansted Fluid Power Ltd, Essex, UK)<br>200 MPa, 120L/h, inlet T°C 6°C, outlet T°C 20°C	No changes in basic enological parameters of the must<br>Complete inactivation of yeasts, molds and lactic acids bacteria; residual total bacteria<br>Better implantation on fermentation starters<br>No significant changes in sensory perception	[262]
Cabernet Sauvignon	300 MPa, 60 L/h, inlet T°C 4°C, outlet T°C 15°C	Reduction of colloidal particle size<br>Complete microbial inactivation<br>No significant impact on basic enological parameters<br>Increased antioxidant activity of must and wine, lower color intensity but higher stability after O2 exposure, in absence of SO2<br>Higher total polyphenols and higher concentration of acetylated anthocyanins<br>Slight increase in total esters, lower concentration of higher alcohols<br>Better aroma quality and global sensory perception	[266]
Hondarribi zuri (white)	300 Mpa, 150 L/h, inlet T°C 20°C, outlet T°C 25°C	Complete yeasts and bacteria inactivation<br>Higher extraction of amino acids and ammonia<br>Reduction of PPO activity, better antioxidant activity of must<br>Higher hydroxycinnamic acids, hue and TPI similar to SO2<br>Lower higher alcohols, and increased esters concentration<br>Improved sensory profile, with fruiter notes	[265]
Muscat of Alexandria	300 Mpa, 60 L/h, inlet T°C 8°C, outlet T°C 15°C	Complete inactivation of native microorganisms<br>Higher concentration of sugars, ammonia, amino nitrogen in must<br>Reduction of mean colloidal particle size<br>Lower enzymatic browning due to PPO inactivation<br>Increased antioxidant activity of must<br>Reduced protein instability	[267]

		Slight changes in volatile compounds, no impact on sensory properties	
Verdejo blanco	300 Mpa, 60 L/h, inlet T°C 8°C, outlet T°C 15°C	More homogeneous and regular size of colloids<br>Complete native yeasts inactivation<br>Higher color hue, also depending on the strains<br>Lower concentration of fermentative volatile compounds, higher concentration of varietal thiols	[263]
Tempranillo	300 Mpa, 60 L/h, inlet T°C 23°C-25°C, outlet T°C 13°C-15°C	Complete native yeasts inactivation<br>No modification in the enological composition of must<br>Higher concentration of total anthocyanins, TPI, chroma, and lower hue, also depending on the strains<br>Lower volatile acids, esters and higher alcohols	[264]
Microwaves			
Variety	Operating Conditions	Results	Ref.
Cabernet Sauvignon	700W, 12 min (3 cycles of 4 min), 40°C	Reduction of maceration time (72h)<br>Total polyphenol index, total tannins and anthocyanins, formation of more stable pigments, higher color intensity	[274]
Cabernet Sauvignon	700 W, 12 min (3 cycles of 4 min), 40°C	Better fermentation kinetics<br>Lower aldehydes and higher concentration of C6 alcohols, terpenes, norisoprenoids and benzenic compounds in must and wine; greater concentration of higher alcohols and acetate esters in wines; better sensory profile	[287]
Cabernet Sauvignon<br><br>Model solution	100-900 W, 30°C-70 °C, treatment time from 2 to 16 min<br>100-900 W, 30°C-70 °C, treatment time from 1 to 9 min	Reduction of PPO 39.58% (500 W, 50°C, 8 min)<br><br>Enhancement of β-glucosidase 12.54% 700W, 48°C, 7 min)	[288,289]
Pinot noir	1150 W, 4 min (cycles of 1 min), 70°C held for 1 h<br>1150 W, 4 min (cycles of 2 min, 1 min, 15-40s), 70°C held for 10 min	Higher juice extraction, higher extraction of yeast assimilable nitrogen<br>Inactivation of total native yeasts<br>Better fermentative kinetics<br>Higher concentration of total phenolics, tannins, anthocyanins, nonbleachable pigments, higher color intensity and stability even after aging (up 18 months)	[275,279,280]
Hamburg Muscat<br><br>Dornfelder	600 W, 2 min for 3 cycles, 85°C-104°C<br><br>1200 W, 8 min, 80°C	Better color parameters<br>Higher extraction of anthocyanins, total phenolic compounds, higher antioxidant capacity of musts and wines	[276,277]
Merlot, Cabernet Sauvignon, Malbec, Syrah, Nebbiolo, Pinot Noir	1200 W, 10 min, 40°C	No effect on basic enological parameters<br>Higher content of anthocyanins, tannins, total phenolics, polymeric pigments, better color evolution, also depending on harvest time, grape variety, vinification and harvest<br>Better sensory profile in Sirah and Cabernet Sauvignon wines	[281,282,284]
Bonarda	7600 W, 15 min, 45–50°C	Slight changes in basic enological parameters<br>Higher concentration of total phenols, tannins, anthocyanins, pigments<br>Higher extraction of grapes polysaccharides<br>Slight modification of wine volatile profile, lower decrease in terpenes and esters content	[283]
Ohmic heating			
Variety	Operating Conditions	Results	Ref.
Vinhão pomace	as a pre-treatment before solvent extraction<br>Frequency 25 kHz, electric field 30 V/cm, 100°C, 13 s	Higher extraction yield of bioactive compounds (total phenolics, ascorbic acid, anthocyanins), with antioxidant and antimicrobial activities	[295]
Grape juice	Frequency 50 Hz, electric field 15, 20, 25 and 30 V/cm.	Lowering of heating rate, processing time and energy consumption<br>Better sensory parameters of juice (color, viscosity, flavor, aroma)	[293]
Cabernet Sauvignon pomace	Electric field 100-800 V/cm, time 0.6 s to 120 s	Higher cell permeabilitazion at higher voltage<br>Higher efficiency in extraction of polyphenols at higher voltage, enhanced in hydroalcoholic solution	[297]
Vinho Verde skins	Frequency 25 kHz, electric field 16 and 80 V/cm, 100°C	Lowering of processing time and energy input consumption<br>Higher extraction of soluble solids, total polyphenol content and anthocyanins	[295]

Grape by-products cv. Vinhão and Loureiro	Frequency 25 kHz, electric field 15 V/cm and 30 V/cm	Higher extraction of phenolic compounds, proteins, insoluble fibers; extracts with higher antioxidant activity	[296]
Cornelian cherry	as a pre-treatment before ultrasound extraction or as extraction method<br>Electric field 20, 30, and 40 V/cm, holding time 1 to 20 min	Increase in extraction yield of total phenolic compounds, total monomeric anthocyanins, soluble solids; extracts with higher antioxidant activity	[299]
Barbera, Aglianico	Frequency 25 kHz, electric field 55 V/cm, time 60 s and 90 s, holding time 15 s, 72°C	No effect on fermentation kinetics<br>Higher total polyphenol index and antioxidant power<br>Higher concentration of esters<br>Better sensory profile, less vegetal and fruitier notes	[300]
Red Globe grapes juice pasterization	Frequency 50 Hz, voltage 8 kV	Significant reduction of spoilage microorganisms<br>Significant reduction of PPO activity<br>Better quality of juice for content of ascorbic acid and anthocyanins, but darker color	[252]
Fresh grape juice	60 Hz, electric field from 0 to 82 V/cm at 65°C and from 0 to 87 V/cm at 75°C<br><br>Different electric field (20-30-40 V/cm), different temperature (60°C-90°C), different holding time (5-25 min)	Inactivation of PPO and POD at higher voltage and temperature	[302,304]
